# Considerations for environmental biogeochemistry and food security for aquaculture around Lake Victoria, Kenya

**DOI:** 10.1007/s10653-023-01585-w

**Published:** 2023-06-02

**Authors:** A. L. Marriott, O. F. Osano, T. J. Coffey, O. S. Humphrey, C. O. Ongore, M. J. Watts, C. M. Aura

**Affiliations:** 1 Inorganic Geochemistry, Centre for Environmental Geochemistry, British Geological Survey, Keyworth, Nottingham, NG12 5GG UK; 2 School of Environmental Sciences, University of Eldoret, Eldoret, Kenya; 3 Kenyan Marine Fisheries Research Institution (KMFRI), Kisumu, Kenya; 4 School of Veterinary Medicine and Science, University of Nottingham, Nottingham, UK; 5 Pelagic Ecology Research Group Scottish Oceans Institute, Gatty Marine Laboratory, University of St Andrews, East Sands St Andrews, Scotland, UK

**Keywords:** Environment, Toxic element, Human health risk, Fish, Aquaculture

## Abstract

The impact of population expansion through economic growth and development has been identified as one of the key drivers of both water and sediment contamination from potentially harmful elements (PHEs). This presents a major hazard not only to aquatic ecosystems but local riparian communities and beyond who rely heavily on this natural resource for drinking water and fish—a valuable source of dietary micronutrients and protein. The present study measured biogeochemical concentration of PHEs in water, sediment and fish from locations pooled into four zones within Winam Gulf and Lake Victoria area of Kenya. Captured fish were used as a sentinel receptor of lake health to evaluate potential risks to fisheries and aquaculture food security. In water, concentrations of arsenic (As), cadmium (Cd), chromium (Cr), copper (Cu) and lead (Pb) were observed above the United States Environmental Protection Agency (US EPA) maximum contamination level drinking water guidelines (MCL), with aluminium (Al) observed above the Aquatic Life Criteria in all four zones. Similarly, sediment concentrations in all four zones exceeded the US EPA Effects range low (ERL) threshold guidelines for Cu, nickel (Ni), zinc (Zn) and Pb, with Cu, Zn and Pb classed at moderate contamination levels using the contamination factor. Fish tissue concentrations from the four zones were calculated using recommended daily intakes (RDI) and for PHEs as provisional maximum tolerable intakes (PMTIs) and indicated most macro- and micronutrients were at or below 10% RDI from aquaculture and wild fish, with Se indicating a greater RDI (16–29%) in all the zones. Contributions of PHEs to PMTIs were below threshold guidelines for both aquaculture and wild fish with only Cd, Cr and Pb levels being above the PMTI thresholds. There is a need to assess the long-term effects of persistent anthropogenic PHE input into Winam Gulf and the wider Lake Victoria basin. Continued monitoring of PHEs using both historical and more recent data will enable future management policies to be implemented through improved mitigation strategies to reduce their impact on water quality, fish health and subsequent human health.

**Supplementary Information:**

The online version contains supplementary material available at 10.1007/s10653-023-01585-w.

## Introduction

Increased anthropogenic activities stemming from urban centre developments and growth of mega-cities, land clearance for agricultural farming purposes and industrialisation (including commercial and artisanal mining) are contributing to increased soil erosion. The effects of these activities are increasing the input of potentially harmful elements (PHEs) such as arsenic (As), chromium (Cr), cadmium (Cd), lead (Pb) and mercury (Hg) into aquatic ecosystems (Mataba et al., 2016; Mdegela et al., 2009; Qian et al., 2020). Furthermore, increased inputs of raw sewage and industrial effluent, which is often poorly regulated combined with agrochemicals in runoff from intensive agricultural farming practices, have added to the input of these contaminants into the surrounding watersheds and lakes (Mataba et al., 2016; Qian et al., 2020).

A number of persistent metals in the aquatic environment adsorb to suspended particles and settle-out on the bottom sediment (Qian et al., 2020). In this form, they are considered inert or stable due to their hydrophobicity, thus reducing the potential toxicity to the aquatic ecosystem (Gong et al., 2021; Qian et al., 2020). However, their accumulation during this process could increase the long-term potential risk should they become re-suspended and remobilised into the surrounding water column through chemical processes or physical disturbances (Gong et al., 2021; Qian et al., 2020). These re-suspended sediment-associated contaminants could further increase their potential risk to the aquatic ecosystem as a secondary source of metal contamination. Furthermore, these chemical transformations could subsequently increase the “bioavailability” of these metals through their uptake by benthic fauna and predation from higher trophic level feeders (Eggleton & Thomas, 2004; Mataba et al., 2016; Nel et al., 2015; Qian et al., 2020; Xia et al., 2019). The combination of catchment contaminants and remobilisation of adsorbed benthic contaminants portends health risks of the PHE to exposed fauna with subsequent potential to bioaccumulation and biomagnification through the aquatic ecosystem food chain (Marriott et al., 2019; Mataba et al., 2016; Qian et al., 2020). This presents a health risk to both the aquatic environment and the local communities and beyond who utilise and rely on these resources as a major source of fish protein and mineral nutrients (Campbell et al., 2003; Connell et al., 1999; Qian et al., 2020).

Kenya’s fishing community is mostly centred on Winam Gulf in the Kenyan portion of Lake Victoria and supports an export industry worth £60 million/year in fish production (FAO, 2015; Odoli et al., 2019) with the fisheries sector employing in excess of 2 million people (Odoli et al., 2019). At 68, 000 km^2^, Lake Victoria is the world’s largest tropical freshwater lake, although relatively shallow has a maximum depth of 69 m at its deepest point. The Lake plays a vital role as a major nutritional food resource, a supply for domestic drinking water and agricultural irrigation for the riparian communities living around the lake shores (Campbell et al., 2003; Okungu et al., 2005; FAO, 2015). Water quality, with regard to the livelihoods of those who rely heavily upon this natural resource, can vary spatially within Winam Gulf compared to the wider Lake Victoria basin (Awange & Obiero, 2006; Omwoma et al., 2014). This is in part due to the localised water circulation patterns within Winam Gulf, its limnology (being shallow throughout its length) and the slow current velocities and a reduced water exchange owing to the narrow water channel at Rusinga that connects Winam Gulf to Lake Victoria. The growth of urban settlements and industries around the Lake shores and inputs from the main rivers (Nyando, Sondu and Awach) entering the Gulf have compounded the effect of increased pollutants into the water leading to increased turbidity and eutrophication (Campbell et al., 2003; Okungu et al., 2005; Okely et al., 2010; Nyamweya et al., 2016; May et al., 2021). Periodic proliferation of water hyacinth has become the hallmark of the long-term changes in the environment and ecology of Lake Victoria with notable effects on the commercial and subsistence fisheries (Ongore et al., 2018; May et al., 2022), which are potentially exacerbated by unsustainable aquaculture practices.

With ever-increasing numbers of consumers, there are currently 40 million people reliant on this valuable food resource for nutrition and subsistence, with demand for fish outstripping fish production. Concerns over declining fish stocks due to their over-exploitation, habitat changes and long-term environmental degradation in the aquatic environment have contributed to food insecurity and malnutrition in Winam Gulf and Lake Victoria basin of Kenya (Aura et al., 2018; Kundu et al., 2017). To compensate for fish demand, aquaculture is seen as a route to the eradication of hunger and poverty (World Bank—Aura et al., 2018) by utilising non-native fish species such as the fast-growing Nile tilapia (*Oreochromis niloticus*). As a species, *O. niloticus* requires minimal maintenance in the form of fish husbandry and is suitable for farming within aquaculture cages (of various dimensions) in Winam Gulf and in the wider Lake Victoria basin (Aura et al., 2018).

The aim of this study was to assess the implications for sustainable aquaculture when faced with increasing inputs of PHE on the aquatic environment of Winam Gulf and the players in the aquaculture and fisheries value chain including the local communities. The objectives to achieve this aim were: (1) measure the aquatic biogeochemistry resulting from lake activities and inputs e.g. water and sediment matrices; (2) measure elemental content of wild and aquaculture fish as a receptor and sentinel species of lake health; (3) evaluate the potential risks to fisheries productivity and food security; and (4) assess food safety using fish as a potential source of essential micronutrients for healthy dietary consumption. These will form the facets of sustainable aquaculture as envisaged by the Food and Agriculture Organization of the United Nation’s concept and framework of Sustainable Food Systems which considers the economic, environmental and social impacts of food production systems (FAO, 2014).

## Materials and methods

### Study sites

**Fig. 1:**
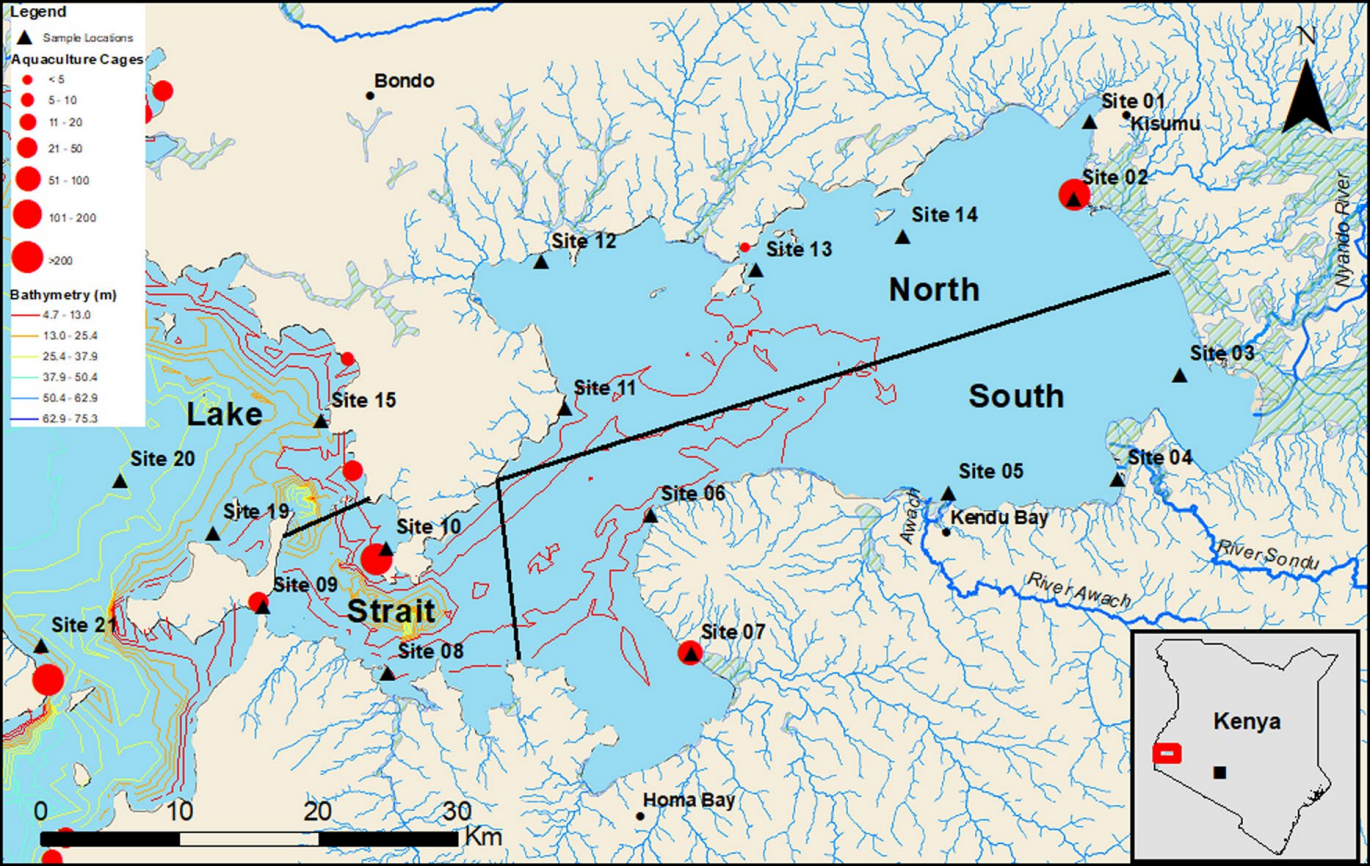
Locations of the sampling sites (triangle) and the boundaries for the four zones comprising of the North: sites 1, 2, 11, 12, 13, 14, South: sites 3, 4, 5, 6, 7, Strait: sites 8, 9, 10 and open Lake: sites 15, 19, 20 and 21 within Winam Gulf and Lake Victoria, Kenya. Cage culture areas are indicated by a red circle. Contains Lake Victoria bathymetry data, available under CC0 1.0 (Hamilton et al., 2016)

The field study and site investigation was conducted onboard the research vessel (RV) Uvumbuzi from the Kenya Marine and Fisheries Research Institute (KMFRI), Kisumu in the Winam Gulf catchment and the wider Lake Victoria basin (Kenyan occupied portion). Field collections were conducted over a two-year period from 2018 to 2019. Two collections were conducted each year during the month of May (wet season), comprising of 12 locations (2018) and 14 locations (2019) and during October/November (semi-dry season) comprising of 14 locations (2018) and 18 locations (2019). The sample sites were chosen for their proximity to: (1) aquaculture “cage” sites; (2) local community beaches; and (3) sources of major river out-flows (Fig. 1).

Due to the narrow Rusinga Channel (flow rate 10–50 cm s^−1^) and the recent re-opening of the Mbita Channel in July 2017 (the channel was previously closed by a causeway built in 1983, (Guya et al., 2017; Simiyu et al., 2022), Winam Gulf and the wider Lake Victoria basin have been defined as separate water bodies (Awange & Obiero, 2006; Omwoma et al., 2014). To account for the collection of samples over a two-year period, possible seasonal effects and for sample collection parameters such as variability within the water column (e.g. samples collected near the surface and at depth), sites where more than one sample had been collected over the two-year survey period were combined to form a composite sample. Additionally, the use of a composite sample enabled an approximation of the chemical composition of the wild and cage fish tissue with regard their natural movement patterns through vertical and horizontal movement within the water column. The locations for the composite samples were then split into four separate zones within the Winam Gulf and the Lake basin, defined as, North: encompassing Kisumu, Dunga (and cages), Ogal, Asat (and cages) and Asembo Bay (and cages), South: encompassing Nyando, Chuowe, Rakwaro, Sondu, Kendu Bay, Mainuga and Homa Bay), the Strait: encompassing Lwanda K’otieno, Mbita), and the open Lake: including areas, Ngodhe, Bridge Island and Mfangano and Mbeo Island (see Fig. 1 and caption).

### Sample collection and preparation

Water samples were collected at each site from one meter below the surface and one meter above the maximum depth (avoiding mixing of the sediments and contamination from surface detritus/algae and sediment/benthos) using a Niskin water sampler. Each site (except May 2018, one station was sampled at each site) followed the same pattern of three stations: offshore, mid-shore and near-shore to address possible spatial chemical changes (see map Fig. 1). Water samples were filtered using a 0.45 µm cellulose syringe filter into 30 mL individually labelled Nalgene LDPE bottles and stored in the on-board refrigerator until arrival at the local laboratory where they were refrigerated until transportation to British Geological Survey (BGS) for analysis. Chemical and physiological water parameters were measured in the field using a Hanna HI-9829 Multiparameter meter for: column depth (m), temperature (°C), dissolved oxygen (DO, mg L^−1^) and pH. Water transparency was measured as Secchi depth (photic depth) using a standard Secchi disk of 20 cm diameter.

Lake sediment samples were collected at each sampling site (in the same locations of the water samples) using a two litre Van Veen grab sampler. Sediments were then sealed into individually labelled polyethylene bags and frozen for transport back to the University of Eldoret laboratory, where the individual sediments were freeze-dried prior to transportation to the BGS. Upon arrival at the BGS laboratories, the freeze-dried sediments were sieved to < 2.0 mm to remove large detritus/rocks using a non-metallic sieve and stored in new individually labelled polyethylene bags prior to acid digestion.

Fish in reared “cage” aquaculture are of the species *O*. *niloticus* (Nile tilapia) which account for approximately 80% of aquaculture production within Winam Gulf and Lake Victoria (Munguti et al., 2017). Between two and four fish with a length range of 16 to 34 cm (total length) were collected from each cage. Wild-sourced fish of the same species and similar length range (15.5 to 33 cm—total length) were collected (where possible) from both local fishermen and beach markets within the location of the aquaculture cages to represent wild food sources. Muscle tissue taken from the caged and wild tilapia was placed into individually labelled clean polyethylene bags and immediately frozen at − 20 °C in the on-board freezers. Upon arrival at the University of Eldoret, the fish muscle tissue was freeze dried and the dry weight (dw) tissue stored in clean polyethylene bags for transportation to the BGS. Upon arrival at BGS, approximately 5 g of fish muscle tissue was ground to a fine powder using a coffee grinder and stored in a clean polyethylene bag in preparation for acid dissolution.

Lake waters were acidified with 1% HNO_3_ & 0.5% HCl for trace metal analysis (Agilent: 8900 ICP-MS/MS). An aliquot of un-acidified water was retained to determine anion concentrations (Dionex: ICS5000), dissolved organic carbon (Shimadzu: TOC-V) and pH/alkalinity (HACH: TIM865) autotitrator. Lake sediments (0.25 g) were dissolved as described by Marriott et al. (2019) and Watts et al. (2017) for a wide range of major and trace elements in a mixed acid solution (5% HNO_3_: 3 mL, 50% HNO_3_: 2 mL, HNO_3_: 3 mL, HF: 2. 5 mL, HClO_4_: 1 mL) on a heat block. Similarly, fish tissue samples (0.25 g, dw) were digested using a mixed acid solution (HNO_3_: 10 mL, H_2_O_2_: 1 mL) in a closed vessel microwave heating system (MARS Xpress, CEMMarriott et al., 2019; Zieritz et al., 2018). Certified reference materials (CRMs) were prepared in the same way as the sediments and fish tissue samples and were simultaneously analysed alongside for quality control. The CRM’s used were: marine/estuarine sediment (MESS-4; PACS-2), Great Lakes sediment (TH-2), Humber River sediment (HR-1), fish muscle (ERM-BB422).

### Assessment methods

Multi-elemental analyses for major and minor trace elements were measured on an inductively-coupled plasma mass spectrometer (ICP-MS) (Agilent: 8900 ICP-MS/MS) as previously described (Watts et al., 2019). Total mercury analysis for fish muscle tissue (0.05 g dw) and lake sediments (0.05 g dw) was measured using a direct mercury analyser (DMA-80, Milestone Inc.). Samples were weighed into individually pre-cleaned (heated to 550 °C for 5 min) nickel weighing boats, and thermally decomposed (using O_2_ rich furnace) at 650 °C and quantifiably measured through atomic absorption (Marriott et al., 2019). The CRMs analysed alongside sediment samples (MESS-4; PACS-2, TH-2, HR-1 and BCR-627) and fish tissue (ERM-BB422). The analytical limits of detection (LOD) were calculated as 3 × the standard deviation of run or digest blanks. Certified reference material data are summarised in Supplementary Tables 1d & 1e; the recovery for the CRMs analysed for Hg on DMA-80 for fish tissue was 98% (ERM-BB422) with sediment between 81 and 117% (MESS-3, MESS-4, PACS-2, TH-2, HR-1). Recoveries for the CRMs (where applicable) analysed on the ICP-MS were between 81 and 113% for sediment (MESS-4) and 105 to 149% for fish tissue (ERM-BB422, BCR-627). The analytical LOD is summarised in the Supplementary Tables 1a, 1b & 1c.

Assessment of the water quality guidelines for the four zones North, South, Strait and Lake was determined using the United States Environmental Protection Agency guidelines (US EPA, 2009), which indicate the water quality thresholds for the Human Health Criteria (HHC) and the National Primary Drinking Water maximum contamination level regulations (MCL) for the PHEs listed (where available). Thresholds were also assessed using the National recommended Aquatic Life Criteria for the criterion continuous concentration (CCC) and the criterion maximum concentration (CMC) to allow comparisons of water quality for aquatic life (US EPA, 2009; URL_1).

Similarly, the assessment of sediment quality guideline threshold limits for the same four zones was determined using the US EPA guidelines (US EPA 1999, 2002) using the effects range low (ERL) guideline at which a biological effect is observed in the aquatic environment and the sediment quality guidelines for freshwater ecosystems (MacDonald, 2000).

### Contamination factor

The contamination status of sediments from the Winam Gulf and the wider Lake Victoria basin was determined using the mean concentrations of metals within the sediment (dw) compared with the average shale concentrations for unpolluted sediments. The CF was calculated as follows:

1 $${\text{CF}} = \frac{{{\text{CS}} - {\text{Mean}}\;{\text{concentration}}\;{\text{of}}\;{\text{element}}}}{{{\text{CB}} - {\text{Average}}\;{\text{metal}}\;{\text{shale}}\;{\text{concentrations}}}}$$

where CS refers to the concentrations of heavy metal in the sediment samples and CB refers to the world average geochemical shale values as shown in Turekian and Wedepohl (1961). Contamination factors are classified in four stages, where CF < 1 indicates a low contamination probability (Stage 1); 1 ≤ CF < 3 moderate contamination probability (Stage 2); 3 ≤ CF < 6 considerable contamination probability (Stage 3) and CF ≥ 6 very high contamination probability (Stage 4) (Hakanson, 1980).

### Geoaccumulation index

The I_geo_ is an estimation of the pollution from metals within the sediment (Müller, 1969, 1979, 1986). The geoaccumulation index is calculated as follows:

2 $$I_{{{\text{geo}}}} = \log_{2} \left( {\frac{{C_{n} }}{{B_{n} \times 1.5}}} \right)$$

where C_*n*_ is the measured concentration of the metal *n* within the sediment, *B*_*n*_ is the geochemical shale background value for the metal *n* from Turekian and Wedepohl (1961) and the constant 1.5 allows for natural lithological variations in the geochemical background of the values and to detect negligible anthropogenic impacts. Descriptions for the *I*_geo_ index (defined by Müller, 1979, 1986) using seven classes are shown in Table 1.

**Table 1:** *I*_geo_ evaluation criteria to define sediment quality using the estimation of pollution from metals within the sediment (defined by Müller, 1979, 1986 and adapted from Singh et al., 2017)

*I*_geo_	Class	Geoaccumulation index
		Sediment quality
≤ 0	0	Uncontaminated
> 0 but < 1	1	Uncontaminated to moderately contaminated
> 1 but < 2	2	Moderately contaminated
> 2 but < 3	3	Moderately–heavily contaminated
> 3 but < 4	4	Heavily contaminated
> 4 but < 5	5	Heavily to extremely contaminated
≥ 5	6	Extremely contaminated

### Bioaccumulation factor determination

The assessment of bioaccumulation factors (BAF), (Arnot & Gobas, 2006) from the element concentrations analysed in the water and dried fish tissue was determined from the ratio of aquaculture and wild caught fish (Nile tilapia: *O*. *niloticus*) to fresh water during equilibrium (Opperhuizen, 1991), and the ratio of fish to sediments (BSAF) using (d*w*) (Alam et al., 2012). The following equations were used:

3 $${\text{BAF}} = {\text{Cn}}_{{{\text{fish}}}} /{\text{Cn}}_{{{\text{water}}}}$$

4 $${\text{BSAF}} = {\text{Cn}}_{{{\text{fish}}}} \left( {{\text{d}}w} \right)/{\text{Cn}}_{{{\text{sediments}}}} \left( {{\text{d}}w} \right)$$

where Cn_fish_ is the concentrations of heavy metal in fish, Cn_water_ is the concentrations of heavy metals in water and Cn_sediments_ are the heavy metal concentrations in sediments. In their review, Arnot and Gobas (2006) defined the categories for metal accumulation as BAF < 1000—no probability of bioaccumulation; BAF > 1000 < 5000—bioaccumulative; BAF > 5000—extremely bioaccumulative.

Using an adapted method proposed by Dallinger et al. (1993), Thomann et al. (1995) calculated metal concentrations in sediment and marine bivalves, where the biota-sediment accumulation factors (BSAF) were defined as BSAF < 1 deconcentrator; BASF > 1 < 2 microconcentrator; BSAF > 2 macroconcentrator. The above calculations were used for this study to assess possible metal accumulation from sediment uptake and associated organic compounds into the tissues of biological receptors (Dallinger, 1993; Alam et al., 2012; Jayaprakash et al., 2015) and to represent the dynamic feeding niche (trophic flexibility) of *O*. *niloticus* within the water column (Njiru et al., 2004; Oso et al., 2006).

### Estimates of nutrient and PHE uptake

Fish tissue weights were converted from dw to ww values using a conversion factor of 0.2 assuming an average water content of approximately 80% (Zieritz et al., 2018; Marriott et al., 2019; see Cressona et al., 2017 on species effect) and applied for the measured recommended daily intake (RDI) and provisional maximum tolerable intakes (PMTI) values referred to in the present study.

Dietary ingestion (DI) rates of macro- and micronutrients from fish were calculated based on the quantity of fish consumed as a 100 g serving (ww) using Eq. 4. The values were then compared to the published RDI values for an adolescent male > 19 years of age (NAS, 2011) and expressed as a percentage.

5 $${\text{DI}}\;{\text{from}}\;{\text{fish}} = {\text{Concentration}}\;{\text{of}}\;{\text{element }}\left( {\frac{{{\text{mg}}}}{{\text{g}}}} \right) \times {\text{Quantity}}\;{\text{of}}\;{\text{consumed}}\;{\text{fish}}\;\left( {\text{g}} \right)$$

PMTIs (JECFA, 2020; URL_2; WHO, 1996; for Ni) for the indicated PHEs were calculated based on the quantities of consumed fish from a 100 g serving (ww) and were expressed relative to body weight using an average Kenyan body weight of 56.265 kg (Speedy, 2003; Walpole et al., 2012), and as such, the total intake of PHEs was corrected for body mass using Eq. 5.

6 $${\text{DI}}\;{\text{of}}\;{\text{PHEs}}\;{\text{in}}\;{\text{fish}}\;\left( {{\text{mg}}/{\text{kg}}\;{\text{bw}}} \right) = \frac{{{\text{Concentration}}\;{\text{of}}\;{\text{element }}\left( {\frac{{{\text{mg}}}}{{\text{g}}}} \right) \times {\text{Quantity}}\;{\text{of}}\;{\text{consumed}}\;{\text{fish}}\;\left( {\text{g}} \right)}}{{{\text{Total}}\;{\text{body}}\;{\text{mass}}\;\left( {{\text{kg}}} \right)}}$$

## Results

### Trace elements in Lake waters

**Fig. 2:**
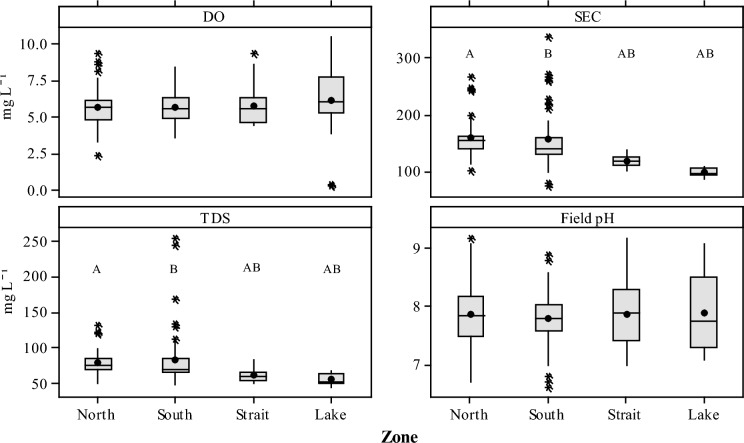
Boxplots of physico-chemical properties taken from Lake water grouped by zone (Northern, Southern, Strait, and Lake). Mean values are indicated by the circle and horizontal bar, with 25th and 75th percentiles indicated by whiskers. Outliers greater than 1.5 times the interquartile range are indicated by an asterix (*). Letters above the boxes indicate statistically significant differences between each of the zones (*P* < 0.05) using a post hoc Tukey test. DO—dissolved oxygen, TDS—total dissolved solids, SEC—electrical conductivity and field pH

The physico-chemical measurements and summary statistics of the waters taken in the four zones from Winam Gulf and Lake Victoria are shown in Table 2, with specific measurements indicated in Fig. 2. The sampled mean water conductivity (SEC) measurements from inside Winam Gulf found zones North and South significantly greater (158 µS cm^−1^ and 157 µS cm^−1^, respectively; *P* < 0.05 Tukey’s pairwise comparisons) than the Strait and Lake zones (119 µS cm^−1^ and 98 µS cm^−1^, respectively). Similarly, total dissolved solids (TDS) measured using a multimeter probe (Hanna instruments) were also found to be significantly greater (*P* < 0.05) in the water from the North and South zones (81 mg L^−1^ and 84 mg L^−1^, respectively) compared to the Strait and Lake zones (62 mg L^−1^ and 57 mg L^−1^, respectively). Additionally, measurements for both DO and field pH were observed to be slightly higher in the Lake and Strait zones when compared to both the North and South zones (Fig. 2); however, these measurements were not significantly different.

**Table 2:** Summary statistics of the chemical water parameters from the survey zones shown in Fig. 1

	Depth^*^	Temperature*	DO*	SEC*	TDS*	pH*	HCO_3_^−^	Cl^−^	SO_4_^2−^	NO_3_^−^	F^−^	DOC
	m	°C	mg L^−1^	µS cm^−1^	mg L^−1^		mg L^−1^	mg L^−1^	mg L^−1^	mg L^−1^	mg L^−1^	mg L^−1^
*North*												
Mean	2.1	27	5.6	158	81	7.9	71	5.1	1.8	0.19	0.52	4.0
SD	1.4	1.2	1.1	31	16	0.55	8.1	1.4	0.36	0.35	0.05	1.5
Max	5.0	30	9.3	265	133	9.2	79	8.7	3.5	2.4	0.59	16.7
Min	< 0.001	23	2.3	101	51	6.7	29	1.8	0.58	0.03	0.24	1.9
*South*												
Mean	1.7	27	5.6	157	84	7.8	74	4.7	1.7	0.69	0.51	4.4
SD	1.9	1.9	1.1	49	37	0.44	27	1.8	0.80	1.4	0.11	3.0
Max	9.0	33	8.4	337	256	8.9	188	11	7.0	8.1	1.0	26.4
Min	< 0.001	21	3.6	73	48	6.6	16	1.5	0.58	0.03	0.20	1.7
*Straits*												
Mean	2.9	26	5.7	119	62	7.9	57	3.9	0.90	0.16	0.37	2.9
SD	2.9	1.5	1.1	9.0	8.6	0.5	8.2	0.53	0.23	0.24	0.05	0.57
Max	18	28	9.3	137	84	9.2	7	4.8	1.3	0.94	0.48	5.7
Min	< 0.001	22	4.4	101	51	7.0	32	1.9	0.38	0.03	0.25	2.1
*Lake*												
Mean	7.5	25	6.1	98	57	7.9	40	2.9	0.42	0.08	0.26	3.2
SD	8.3	2.1	2.6	5.7	7.9	0.66	14	1.1	0.20	0.15	0.05	2.7
Max	26	28	11	107	68	9.1	56	4.5	0.89	0.64	0.32	14.1
Min	1.0	22	0.18	87	44	7.1	12	0.57	0.07	0.03	0.15	1.1

Parameters measured in the field are denoted by *. All other parameters were measured in the laboratory at BGS

DO, dissolved oxygen; SEC, conductivity; TDS, total dissolved solids; HCO_3_^−^ total alkalinity; DOC, dissolved organic carbon; SD ± 1 standard deviation

**Fig. 3:**
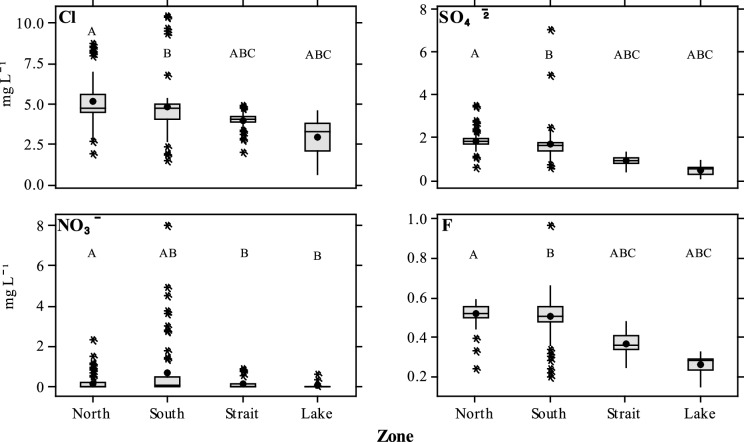
Boxplots of major (Cl^−^, SO_4_^2−^, NO_3_^−^) and one minor (F^−^) anions taken from Lake water grouped by zone (North, South, Strait and Lake). Mean values are indicated by the circle and horizontal bar, with 25th and 75th percentiles indicated by whiskers. Outliers greater than 1.5 times the interquartile range are indicated by an asterix (*). Letters above the boxes indicate statistically significant differences between each of the zones (*P* < 0.05) using a post hoc Tukey test

Levels of Cl^−^, SO_4_^2−^, NO_3_^−^ and F^−^ were significantly higher (*P* < 0.05) in the North (mean of 5.1, 1.8, 0.2 and 0.5 mg L^−1^, respectively) and South zones (mean of 4.7, 1.7, 0.7 and 0.5 mg L^−1^, respectively) when compared to Strait (mean of 3.9, 0.9, 0.2 and 0.4 mg L^−1^, respectively) and Lake zones (mean of 2.9, 0.4, 0.1 and 0.3 mg L^−1^, respectively) (Table 2). Differences for Cl^−^, SO_4_^2−^ and F^−^ were at the significant level (*P* < 0.05 (Fig. 3) using a post hoc Tukey test when comparing the North and South zones to the Strait and Lake zones. Nitrate was also observed to be significantly different between zones (*P* < 0.05) specifically concentrations observed between the North and South and when comparing the South to the two zones the Strait and Lake.

The descriptive statistics for selected PHEs measured in water can be found in the supplementary table (Suppl. Table 2) with water quality guidelines published by the US EPA (US EPA, 2009). The guidelines used indicate the water quality thresholds for the Human Health Criteria (HHC) and the National Primary Drinking Water regulations (MCL) for the PHEs listed (where available). The thresholds using the national recommended Aquatic Life Criteria are also shown for the criterion continuous concentration (CCC) and the criterion maximum concentration (CMC) to allow comparisons of water quality for aquatic life (US EPA, 2009; URL_1).

The PHE mean concentrations for five elements As, Cd, Cr, Cu and Pb were found to be above the US EPA (2009) MCL for all of the four zones with the exception of Cu (South, Straits and Lake) and Cr (Lake) (Suppl. Table 2). The concentrations of As were observed above the HHC threshold stipulated by the US EPA for national primary drinking water regulations (US EPA, 2009; URL_1). However, PHE concentrations were found to be below the US EPA guidelines when assessing the two water quality guidelines against the CCC and the CMC (Suppl. Table 2), with the exception of Al, which exceeded the recommended CCC for three of the four zones North, South and Strait by a factor of 9, 5 and 3, respectively. Additionally, the North zone also exceeded the CMC (CMC = 750 µg L^−1^) for Al (851 µg L^−1^). Levels of Cu and Pb exceeded their respective CCC in the Strait zone (Suppl. Table 2).

**Fig. 4:**
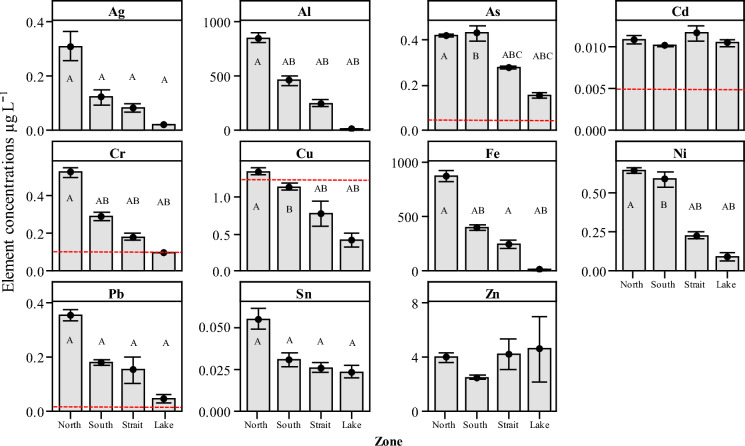
Interval plots of potentially harmful elements in water for the whole survey grouped by zone (North, South, Strait and Open-Lake). Mean values indicated with 95% confidence interval. Dotted lines indicate maximum contamination level (MCL) threshold for clarity. Letters above the boxes indicate statistically significant differences between each of the zones (*P* < 0.05) using a post hoc Tukey test

Interval plots for 11 PHEs from the four sampling zones are indicated in Fig. 4. Using a post hoc Tukey test, no significant differences were observed for two of the elements Cd and Zn (*P* > 0.05). However, for the remaining nine elements significant differences were observed between individual element concentration levels and between zones (Fig. 4). Most notably, levels of Ag between the North zone and the remaining three zones South (*P* = 0.008), Strait (*P* = 0.002), Lake (*P* = 0.009) were significant higher. Significant differences between the North zone and the three remaining zones were also observed for Pb (all *P* < 0.001), and for the Sn (all *P* < 0.05).

### Winam Gulf and Lake sediments

Descriptive statistics for ten selected PHE concentrations determined in the sediment collected from the four zones are presented in Table 3. The levels of Ag, Al, As, Cd, Cr, Cu, Ni, Pb, U and Zn indicated some variability between the four zones when assessing their maximum and minimum concentrations. Most notably, lower Ag and Hg concentrations were found in the North zone (1.1 and 0.17 mg kg^−1^, respectively) when compared to the South, Strait and Lake zones. Conversely, As was higher in the South (13 mg kg^−1^) when compared to the North, Strait and Lake zones. Maximum Pb concentrations were considerably higher in the North and South zones (86 and 105 mg kg^−1^) and decreasing when compared to lower maximum concentrations observed in the Strait and Lake (36 and 23 mg kg^−1^).

**Table 3:** Concentrations of selected potentially harmful elements and their descriptive statistics in Winam Gulf and Lake Victoria sediments

	Ag	Al	As	Cd	Cr	Cu	Fe	Ni	Pb	Sn	Zn	Hg
*SQG*												
TEC	–	–	9.79	0.99	43.4	31.6	–	22.7	35.8	–	121	0.18
PEC	–	–	33	4.98	111	149	–	48.6	128	–	459	1.06
*U.S. EPA*												
ERL	1.0	–	8.2	1.2	**81**	**34**	–	**20.9**	46.7	–	**150**	0.15
ERM	3.7	–	70	9.6	370	270	–	51.6	218	–	410	0.71
*North*												
Mean	0.25	84,508	3.5	0.26	63	**50**	63,633	**35**	24	4.5	**165**	0.05
SD	0.18	16,488	1.4	0.08	29	27	18,862	10	11	1.7	52	0.03
Max	1.1	108,996	5.4	0.52	247	131	125,118	50	86	6.9	262	0.17
Min	0.20	37,076	0.2	0.03	32	1.4	5351	6.1	6.6	0.18	7.0	0.002
*South*												
Mean	0.24	78,034	5.6	0.32	63	**39**	85,626	**40**	34	4.8	**201**	0.03
SD	0.06	18,566	2.8	0.12	22	22	51,057	19	20	1.6	99	0.02
Max	0.47	131,692	13	0.57	109	77	254,282	74	105	8.6	466	0.05
Min	0.20	40,073	0.5	0.08	25	3.8	24,351	7.0	12	0.8	37	0.002
*Straits*												
Mean	0.23	72,887	2.8	0.25	**88**	**74**	83,688	**39**	20	3.8	**173**	0.04
SD	0.05	13,171	0.9	0.06	81	24	15,006	12	5.0	0.5	32	0.01
Max	0.36	85,223	4.2	0.35	386	121	132,332	88	36	4.8	255	0.06
Min	0.20	31,159	0.3	0.09	39	22	52,369	27	9.2	2.5	65	0.004
*Lake*												
Mean	0.20	62,091	3.6	0.20	71	**66**	78,915	**34**	17	3.3	**145**	0.07
SD	0.00	11,194	1.2	0.06	37	22	14,978	2.0	2.9	0.3	15	0.02
Max	0.20	72,549	4.7	0.30	167	109	108,051	37	23	3.7	165	0.10
Min	0.20	37,062	0.9	0.12	44	40	52,048	30	13	2.8	118	0.03

All values are in mg kg^−1^. Samples are measured as dry weight using sediment quality guidelines from MacDonald (2000) PEC—probable effect concentration, and TEC—threshold effects concentration and the US EPA (US EPA, 1999, 2002), ERL—effects range low, ERM—effects range media. Mean values greater than the effect range low (ERL) are highlighted in bold. SD ± 1 standard deviation

**Fig. 5:**
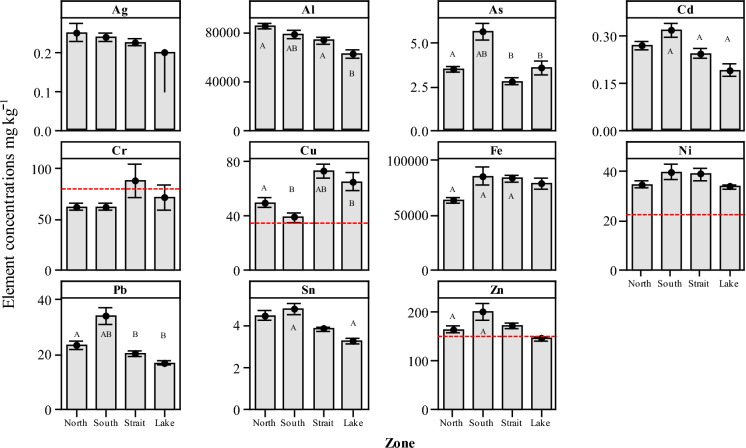
Interval plots of potentially harmful elements in Lake sediments of 11 elements grouped by zone North, South, Strait and Lake. Mean values indicated with 95% confidence interval. Dotted lines indicate ERL threshold for clarity. Letters above the interval plots indicate statistically significant differences between each of the zones (*P* < 0.05) using a post hoc Tukey test

The concentrations of several PHEs in the sediments were found to exceed the guideline threshold limits published by the US EPA (US EPA 1999, 2002) and the sediment quality guidelines for freshwater ecosystems (MacDonald, 2000). Using the US EPA effects range low (ERL) guideline at which a biological effect is observed in the aquatic environment, levels of Cu and Ni were above the ERL values (ERL: Cu 34 and Ni 20.9 mg kg^−1^, respectively) for all four zones (Table 3). Other ERL exceedances were observed for Zn (ERL: 150 mg kg^−1^) in three of the four zones (North, South and the Strait) and for Cr (ERL: 81 mg kg^−1^) in the Strait (Fig. 5). Despite some PHEs being observed above the ERL guidelines, levels of Ag, Cd, Cr and Pb were found not to exceed the threshold in any of the four zones. It was also noted that concentrations of Cr, Cu, Ni and Zn exceeded the US consensus-based threshold effects concentration (TEC) (MacDonald, 2000) in all of the four zones.

Interval plots for sediment concentrations of 11 PHEs observed from the four zones are indicated in Fig. 5. No significant differences were observed for three elements Ag, Cr and Ni (*P* > 0.05) and between the four zones. However, for the remaining eight elements, significant differences were observed between individual element concentration levels and between zones (Fig. 5). Sediment concentrations of As were significantly higher between the North and South zones (*P* < 0.001) and between the South and remaining two zones Strait and Lake (*P* < 0.001 and 0.01, respectively). Similarly, sediment concentrations of Pb were significantly higher between the North and South zones (*P* < 0.001) and between the South the Strait and Lake zones (*P* < 0.001 and 0.004, respectively).

### Contamination indices

Determination of the CFs for sediment quality was measured from the four zones with all of the PHEs exceeding the lowest contamination probability of < 1 for this study (Table 4). In addition, PHEs were observed between the moderate contamination probability factors (1 ≤ CF < 3) for North (Al, Cu, Fe, Pb, Th, Ti and Zn), South (Cd, Fe, Pb, Ti, Zn and U), Strait (Cu, Fe, Mg, Pb, Th, Ti and Zn) and Lake (Ag, Cu, Fe, Th, Ti and Zn). Levels of Ag exceeded the next CF level with a considerable contamination probability (3 ≤ CF < 6) for the North, South and Strait. Only one other PHE, Th was found to have a similar CF of 3 ≤ CF < 6 for the South zone (Table 4).

**Table 4:** Contamination factors for selected element concentrations in the sediment from the four zones sampled in Winam Gulf and Lake Victoria

	Ag	Al	As	Cd	Cr	Cu	Fe	Hg	Mg	Ni	Pb	Sn	Th	Ti	U	Zn
CF	0.07	80,000	13	0.3	90	45	47,200	0.4	15,000	68	20	6	12	4600	3.7	95
North	3.64	1.06	0.27	0.86	0.70	1.11	1.35	0.12	0.52	0.51	1.17	0.75	1.65	1.63	0.90	1.74
South	3.49	0.98	0.43	1.05	0.70	0.87	1.81	0.07	0.51	0.59	1.70	0.80	3.13	1.60	1.41	2.12
Straits	3.22	0.91	0.21	0.82	0.98	1.64	1.77	0.10	1.03	0.57	1.02	0.64	1.44	2.53	0.72	1.82
Lake	2.86	0.78	0.27	0.66	0.79	1.46	1.67	0.17	0.80	0.50	0.85	0.55	1.29	2.18	0.75	1.53

Values are in mg kg^−1^. Values are determined from the International geochemical background shale values as defined in Table 1

Calculations using the Geoaccumulation Index (*I*_geo_ Table 1) for the majority of the PHEs were indicating an I_geo_ value below zero (Table 5). Values below zero were observed for As, Cd, Cr, Cu, Hg, Mg, Ni, Pb and U (*I*_geo_ < 1) indicating that the four zones were uncontaminated by these metals. However, Ag, Mn and Th had positive values with an *I*_geo_ value of moderately contaminated (> 1 but < 2) for the zones North, South and Strait (Ag) and South for both Mn and Th.

**Table 5:** Geoaccumulation Index (*I*_geo_) values are indicted below for selected element concentrations in the sediment from the four zones sampled in Winam Gulf and Lake Victoria

	Ag	Al	As	Cd	Cr	Cu	Fe	Hg	Mg	Mn	Ni	Pb	Sn	Th	Ti	U
*I*_geo_	0.07	80,000	13	0.3	90	45	47,200	0.4	15,000	850	68	20	6	12	4600	3.7
North	1.28	− 0.51	− 2.49	− 0.80	− 1.11	− 0.43	− 0.15	− 3.61	− 1.52	− 0.05	− 1.55	− 0.35	− 1.00	0.14	0.12	− 0.74
South	1.22	− 0.62	− 1.79	− 0.51	− 1.11	− 0.79	0.27	− 4.39	− 1.57	1.25	− 1.36	0.18	− 0.91	1.06	0.09	− 0.09
Straits	1.10	− 0.72	− 2.81	− 0.87	− 0.61	0.12	0.24	− 3.91	− 0.54	0.82	− 1.39	− 0.56	− 1.23	− 0.06	0.75	− 1.06
Lake	0.93	− 0.95	− 2.45	− 1.18	− 0.92	− 0.04	0.16	− 3.18	− 0.91	0.11	− 1.60	− 0.82	− 1.45	− 0.22	0.54	− 1.01

Values are in mg kg^−1^. Values are determined from the International geochemical background shale values as defined in Table 1

### Contamination factor

See Table 4.

### Geoaccumulation index

See Table 5.

### Fish tissue

**Fig. 6:**
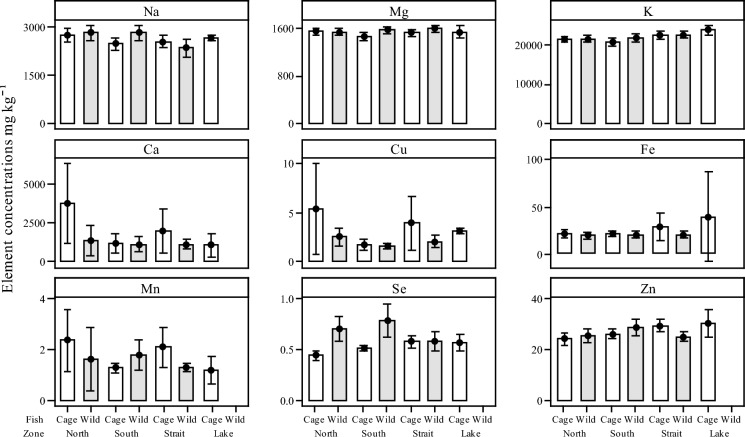
Interval plots of selected elemental concentrations of available macro-/micronutrients in the fish muscle tissue (mg kg^−1^ dry weight) from both caged and wild caught Nile tilapia (*O*. *niloticus*) grouped by zone (North, South, Strait and Lake). Mean values indicated with 95% confidence intervals, the error bars for Fe in the Lake zone are due to small sample numbers for this zone. Data are shown in Suppl. Table 3

The total mean concentrations and their respective standard deviations (SD) of selected essential and non-essential PHEs in muscle tissues from both caged and wild caught Nile tilapia (*O. niloticus*) are defined in (Suppl. Table 3). Interval plots for selected elements indicated as macro- and micronutrients measured in the caged and wild caught fish sampling zones are summarised in Fig. 6. Of the nine elements Na, Mg, K, Ca, Cu, Fe, Mn and Zn, only levels of Na, Zn and Se showed significant differences within the elemental concentration of the fish tissue between caged and wild caught fish and zone of capture. The concentration of Na between wild caught fish in the North zone when compared to wild caught fish from the Straits was significantly higher (*P* = 0.02). Similar Zn concentrations in fish tissue from the caged fish in the south were significantly lower than in the caged fish from the Straits (*P* = 0.007). The concentrations of Se in the fish tissue varied significantly between caged and wild fish and zone. Levels in the caged fish from the North were significantly lower when compared to the wild fish caught from the North and South zones (*P* < 0.001). Similarly, levels of Se in wild caught fish from the North were significantly higher than in cage fish from the South (*P* < 0.04). Concentrations of Se in cage caught fish from the South were significantly lower compared to the wild caught fish from the same zone (*P* = 0.004). Furthermore, levels of Se from wild caught fish from the South were significantly higher when compared to both wild and caged fish from the Strait zone (*P* = 0.02; *P* = 0.04, respectively).

**Fig. 7:**
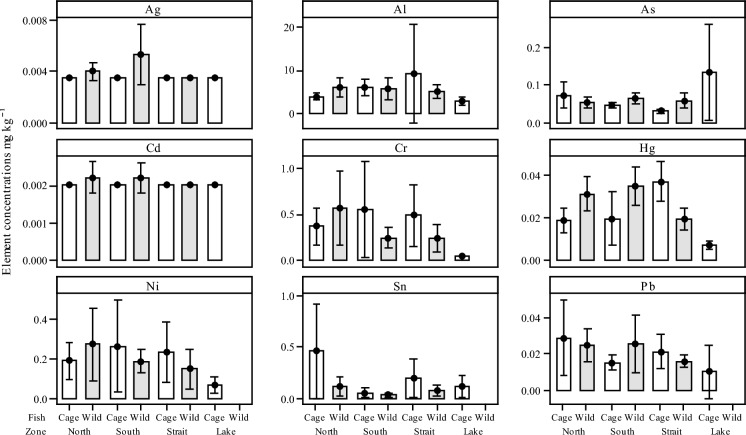
Interval plots of selected potentially harmful element (PHE) concentrations in the fish muscle tissue (mg kg^−1^ dry weight) collected from both caged and wild caught Nile tilapia (*O*. *niloticus*) grouped by zone (North, South, Strait and Lake). Mean values indicated with 95% confidence intervals. Data are shown in Suppl. Table 3

Interval plots for the selected PHEs measured in the tissue from caged and wild caught Nile tilapia (*O*. *niloticus*) for each of the sampling zones are summarised in Fig. 7. Elemental concentrations were assessed for seven elements Al, As, Cr, Hg, Ni, Pb and Sn. Of these, only Hg was observed to have significant differences in concentrations in the fish tissues and between zones. Most notably, levels of Hg between cage fish in the North and cage fish in the Strait (*P* = 0.005), cage fish from the South and the Strait (*P* = 0.04) and concentrations in the tissue of cage fish from the Strait and wild fish from the same zone (*P* = 0.02).

### Bioaccumulation factors of PHEs in Nile tilapia

The bioaccumulation factors (BAF’s) and biota-sediment accumulation factors (BSAF) of Nile tilapia were measured using the fish muscle tissue for both water and sediment calculations, respectively (Suppl. Table 4 and Suppl. Table 5). The values of metals from BAF calculations < 1000 indicate no probability of bioaccumulation; with BAF values > 1000 but < 5000 bioaccumulative and BAF values > 5000 are extremely bioaccumulative. Results from this study shown in Suppl. Table 4 indicated BAF values for both caged and wild tilapia Cn_fish_/Cn_water_ for the PHEs defined were all < 1000 and therefore indicating no probability of accumulation. However, it must be noted that BAF values obtained for K in *O*. *niloticus* in the aquatic environment were found to be > 1000 but < 5000 for the North zone from caged (BAF: 3910) and wild (BAF: 3942) fish, respectively, indicating probable bioaccumulative effects (Suppl. Table 4). Additionally, the South and the Straits had BAF values > 5000 (South—BAF: 5248 and BAF: 5516; Straits—BAF: 6225 and BAF: 6293) for caged and wild tilapia, respectively, indicating extreme bioaccumalative potential. Furthermore, K was observed at twice the BAF value > 5000 (BAF: 13113) in the fish from the Lake zone. Similarly, BAF values for Mg (BAF: 1141) in fish from the Lake zone indicate a possible bioaccumulative effect. The BAF values ascertained from the sediment associated PHEs across the four zones for both caged and wild caught *O*. *niloticus* were below the threshold value of < 1000 and therefore had no probability of bioaccumulation (Suppl. Table 5).

The BSAF parameters as defined from the accumulation of sediment-associated compounds (PHEs) in tissues were calculated for the present study. BSAF values are classified as > 2 (macro-concentrator); < 2 > 1 (micro-concentrator) and < 1 (deconcentrator) for the PHEs defined in Suppl. Table 5. All PHEs in the wild fish with the exception of one site for Hg (South, BSAF: 1.06) were all < 1 and classed as a de-concentrator. However, as observed for BAF values measured in fish tissue and surrounding water, the BASF values for K and Se from both caged and wild fish in the zone indicated in Suppl. Table 5 were classed as potential micro-concentrators (BSAF < 2 > 1).

### Recommended daily intakes and provisional maximum tolerable intakes

**Fig. 8:**
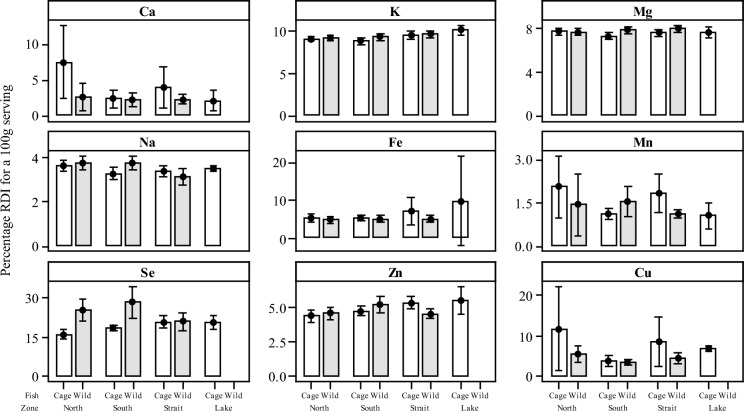
Interval plots of recommended daily intakes (RDI) calculated as a percentage of macro (Ca, K, Na, Mg)- and micro (Cu, Fe, Mn, Se, Zn)-essential elemental contribution (expressed as wet weight) from a 100 g serving of fish from both caged and wild caught Nile tilapia (*O*. *niloticus*) grouped by zone (North, South, Strait and Lake). Mean values indicated with 95% confidence intervals

Estimations for the concentrations of available macro- and micronutrients in a 100 g serving of fish (ww) from cage and wild caught fish are indicated in Table 6 and illustrated in Fig. 8. The RDIs from the present study indicated that consuming a 100 g portion of either the cage cultured or wild fish would provide a minimal of ≤ 10% for the five macronutrients Ca, K, Mg, Na and Fe from each of the four zones. Although low RDIs were observed for both cage and wild fish (RDI: < 10%), the potential contribution of Fe was notably higher in the caged fish from each of the four zones when compared to wild fish caught in the same locations (no wild fish were collected from the Lake zone). Most notably, levels of Fe were higher in caged fish from the Strait and Lake (29–40 mg kg^−1^ dw) when compared to levels in wild fish across three of the four zones (19–20 mg kg^−1^ dw). Equally, the contribution from a 100 g serving of cage cultured or wild tilapia from the four zones for the micronutrients Mn, Zn and Cu was also minimal ≤ 10% RDI, with the exception of Cu in caged cultured fish from North zone (RDI > 12%) (Table 6 and Fig. 8). Although the potential contribution to the RDI of macro-/micronutrients was minimal for both caged and wild caught fish, they were both found to be a good source of Se, with the RDI calculated using a single portion of 100 g of fish consumed accounting for up to 30% of the RDI for a single serving (28.7%). Wild tilapia accounted for higher levels of Se from the four zones (0.58–0.79 mg kg^−1^ dw) and a greater contribution to the RDI (21 to 29%), compared with caged tilapia accounting for lower concentrations (0.45—0.58 mg kg^−1^ dw) and a reduced contribution to the RDI for Se (16–21%).

**Table 6:** Recommended daily intakes (RDI) calculated as a percentage (%) for an adult > 19 years of age using a contribution of 100 g (ww) serving of cage or wild Nile tilapia (*O*. *niloticus*) calculated as an average from each of the four zones: North, South, Strait and Lake

	Ca	K*	Mg	Na*	Fe	Mn*	Se	Zn	Cu
	mg d^−1^	mg d^−1^	mg d^−1^	mg d^−1^	mg d^−1^	mg d^−1^	mg d^−1^	mg d^−1^	mg d^−1^
	1000	4700	400	1500	8.0	2.3	0.055	11.0	0.9
*North*									
Cage	7.5	9.1	7.7	3.7	5.3	2.1	16.2	4.4	11.8
Wild	2.6	9.2	7.7	3.8	4.8	1.4	25.8	4.6	5.5
*South*									
Cage	2.3	8.9	7.3	3.3	5.3	1.1	18.8	4.8	3.8
Wild	2.2	9.3	7.8	3.8	5.0	1.5	28.7	5.3	3.5
*Strait*									
Cage	4.0	9.5	7.6	3.4	7.2	1.8	21.1	5.4	8.6
Wild	2.2	9.6	8.0	3.1	5.0	1.1	21.2	4.6	4.4
*Lake*									
Cage	2.0	10.1	7.7	3.5	9.9	1.0	20.9	5.6	6.7

All data have been rounded up to 1 decimal place for clarity. RDI values from NAS 2011

*Adequate intake as recommended by National Academy of Sciences (USA), 2011

### Provisional maximum tolerable intake

Using Eq. 5, concentrations of PHEs were calculated from a 100 g serving of Nile tilapia (ww) using an adult (> 19 year) and an average Kenyan body weight of 56.26 kg mg/kg bw/d. Data were then assessed against the provisional maximum tolerable intakes (PMTI) guidelines and are shown in Table 7. Contributions of Al, As, Hg, Ni and Sn were all below the PMTI guidelines set by the FAO/WHO, with most zones and fish type having similar values. However, three PHEs Cr, Pb and Cd exceeded the PMTI guidelines (FAO/WHO, 2020) for both cage and wild fish in all four zones. Variations in the PMTI for Cd in each of the four zones were evident when comparing the caged and wild fish, with wild fish having more Cd in the North and South zones. Cage fish also exceeded the PMTI guidelines for Cd in all four zones but had lower concentrations compared to their wild counterparts (Table 7).

**Table 7:** Provisional maximum tolerable intakes (PMTIs) calculated using a daily intake for an adolescent > 19 years of age and an average Kenyan body weight of 56.26 kg mg/kg bw/d, using a contribution of 100 g (ww) serving of cage and wild Nile tilapia (*O*. *niloticus*) calculated as an average from each of the four zones: North, South, Strait and Lake. PMTI values from NAS, 2011, JECFA, 2020 and WHO, 1996

PMTI	Al	As^**#**^	Cr*	Hg	Ni	Pb^**#**^	Sn	Cd
	mg/kg bw/d	mg/kg bw/d	mg/kg bw/d	mg/kg bw/d	mg/kg bw/d	mg/kg bw/d	mg/kg bw/d	mg/kg bw/d
	0.3^2^	0.003	0.035^1^	0.004^2^	0.60^3^	0.003^2^	2.0^2^	0.0008^2^
PTWI	2 mg/kg bw/w^2^	0.0001–0.003^2^	0.025 mg/kg bw/w^2^	0.0016 mg/kg bw/w^2^		0.00002 − 0.003^2^	14 mg/kg bw/w^2^	PTMI 0.025 mg/kg bw/m^2^
*North*								
Cage	0.005	0.0001	**0.2**	0.002	0.0001	**0.003**	0.0001	**0.001**
Wild	0.007	0.0001	**0.3**	0.003	0.0002	**0.003**	0.00002	**0.1**
*South*								
Cage	0.01	0.0001	**0.3**	0.002	0.0002	0.002	0.00001	**0.001**
Wild	0.01	0.0001	**0.1**	0.003	0.0001	**0.003**	0.00001	**0.1**
*Strait*								
Cage	0.01	0.00004	**0.3**	0.003	0.0001	**0.003**	0.00004	**0.001**
Wild	0.01	0.0001	**0.1**	0.002	0.0001	0.002	0.00001	**0.001**
*Lake*								
Cage	0.004	0.0002	**0.03**	0.001	0.00004	0.001	0.00002	**0.001**

*Adequate intake as recommended by National Academy of Sciences (USA), 2011

^**#**^Best Estimate as recommended by Joint FAO/WHO Expert Committee (JECFA), 2020

^1^National Academy of Sciences (USA), 2011

^2^Provisional maximum tolerable intakes (PMTIs) Joint FAO/WHO Expert Committee on Food Additives (JECFA), 2020

^3^World Health Organisation (WHO) 1996. Trace elements in human nutrition (Technical Report Series, No. 532)

## Discussion

Increasing demand for natural resources from land and water through rapid population growth has resulted in increased anthropogenic activities on and around the shores of Winam Gulf and the wider Lake Victoria basin in Kenya. Both terrestrial and aquatic ecosystems have experienced increased activities from changes in land use and subsequent effects from soil erosion and land degradation to the detriment of water quality through pollution and loss of biodiversity. In this study, we assessed the impacts from PHEs such as metal pollution from both anthropogenic and geogenic processes and their potential risks to human health from bioaccumulative effects from the consumption of water and caged and wild caught fish.

### Water physico-chemical parameters

Water physico-chemical measurements from within Winam Gulf from the North and South zones had elevated values for conductivity when compared to the Strait and the open Lake zones (Table 2). Conductivity measurements were higher in Winam Gulf (up to 158 µS cm^−1^) compared to the Straits and Lake zones (98 to 119 µS cm^−1^) similar to findings from a previous study (Ochieng et al., 2006). Although conductivity values measured were below the potable water ranges indicated by the US EPA (50 to 1500 μS cm^−1^ URL_4), variable conductivity levels may indicate changes in water quality and therefore could be attributed to a rise in the influx of surface run off and the rivers feeding into the Winam Gulf (Gikuma-Njuru & Hecky, 2005), such as the River Nyando and River Sondu, and the limited exchange between the two zones compared to the zones closest to the main body of the Lake and the Rusinga Channel (Calamari et al., 1995; Gikuma-Njuru & Hecky, 2005).

Water chemistry measured from the four zones indicated similar traits for Cl^−^, SO_4_^2^ and NO_3_^−^, which had elevated concentrations for the North and South zones compared to the Strait and Lake zones. The mean concentration levels of NO_3_^−^ for the North and South zones were higher (although below threshold guidelines, US EPA, 2009) to those previously reported (Gophen et al., 1995; Lung’ayia et al., 2001; Gikuma-Njuru & Hecky, 2005; LVEMP, 2002; Sitoki et al., 2010; Juma et al., 2014). The trend in nutrient enrichment indicated by Juma et al. (2014) saw a steady increase in the previous two decades, although the present study indicated there has been little change since 2018. Indeed, riverine contributions to Winam Gulf may account for the increased levels of NO_3_^−^, in the form of terrestrial N, found in the present study and may arise from a rapid growth in agricultural practices of land clearance, increased soil erosion and a resuspension of sediment particles in Winam Gulf (Gikuma-Njuru & Hecky, 2005). Similarly, concentrations of Cl^−^ and SO_4_^2^ were at higher levels in the North and South zones when compared to the Straits and Lake, although below threshold guidelines (US EPA, 2009). These could be attributable to natural mineral deposits within the catchment area or possibly from the influence of irrigation and increased anthropogenic activities, such as industrial and domestic discharges (Brandt et al., 2017; Mwamburi, 2003; Oyoo-Okoth et al., 2010). This could be linked to locations where there has been a rapid growth of urban settlements and increases in human activities (Juma et al., 2014; Lung’ayia et al., 2001) such as the city of Kisumu and Asembo Bay (North zone) and towns located in the riparian areas of Kendu Bay and Homa Bay (South zones).

Assessments of the water quality from PHEs in the four zones indicated the mean concentration levels of As, Cd, Cr, Cu and Pb were above US EPA (2009) drinking water guidelines, whereas previous studies by Ongeri et al. (2009) and Ochieng et al. (2006) had As, Cd, Cr, Cu and Pb concentration levels of one and two orders of magnitude higher, respectively, when comparing their data and sampling locations in Winam Gulf to those from the present study. Notably, recent improvements in water quality in the Nyanza gulf have been observed and linked to the opening of the Mbita causeway in May 2017 (Guya et al., 2017; Simiyu et al., 2022). Additionally, average PHE values for the four zones were observed to be below the threshold guidelines stipulated for Aquatic Life Criteria (US EPA, 2009, URL_1), with only Al exceeding this criterion in three of the four zones including the maximum threshold values for the North, South and Strait. Although aluminium occurs naturally within the environment, increased levels observed in these zones may be a result of localised mining, industrial activities and from coal burning power stations and incinerators. The utilisation of the water systems from the Winam Gulf resources has allowed the fluid movement of these PHEs downstream, ultimately concentrating at their end-point at the river mouths (Mwamburi, 2003; Ongeri et al., 2009). This accumulation, whether direct or indirect, has increased their abundance within the surrounding aquatic environments (Bakyayita et al., 2019). Subsequently, this could have serious consequences with regards the use of the vast riverine systems by local communities as a source of drinking water (human or livestock), with further implications for food security where fisheries (aquaculture or wild capture) are utilised, e.g. Dunga cages site 2 (Fig. 1).

### Sediment chemical parameters

Sediment concentrations measured for selected PHEs, Cu, Ni, Zn and Pb in the four zones exceeded some of the US EPA threshold guidelines. This was also evident when assessing the CF for Cu, Zn and Pb measured in the sediments which were at a moderate level of contamination. However, when using the *I*_geo_ index only Ag indicated a high level of contamination in three of the zones, North, South and Strait, with the *I*_geo_ index indicating Ag was having a human-derived impact albeit minimal within the three zones indicated. Conversely, Ag was not observed to be above any US EPA threshold guidelines and moreover was an order of magnitude below the US EPA ERL guideline, and as such would be of a low concern in terms of fish-food safety issues on fish production, consumption and utilisation by the local communities.

In the present study, the spatial variability was evident for PHEs between the four zones and when comparing these data with previous studies (Ochieng et al., 2006; Ongeri et al., 2009; Outa et al., 2020). However, assessing possible changes over a temporal scale is somewhat more challenging. Using previous studies and pooling their individual sites within each of the four zones (where applicable), some assumptions could be inferred with PHEs indicating some stability in one or more of the four zones. Sediment concentrations of Cu in the North, South and Lake zones were relatively consistent and comparable to previous studies from Ochieng et al. (2006) (31.3 mg kg^−1^, 44.0 mg kg^−1^ 47.7 mg kg^−1^, respectively), Ongeri et al. (2009) (50.4 mg kg^−1^, 35.4 mg kg^−1^ and 18.1 mg kg^−1^, respectively) and Outa et al. (2020) (44.2 mg kg^−1^, 26.6 mg kg^−1^ and 46.9 mg kg^−1^, respectively). However, Cu levels in these three zones would suggest a slight increase overall, when compared to the Outa et al. (2020) study. However, the overall Cu levels observed within Winam Gulf have indicated little change for the last 15 years despite the changes in land use and expansion of the population in the surrounding coastal areas such as Kisumu, Kendu Bay, Homa Bay and Asembo Bay.

The concentrations of Pb in the sediments from all four zones were below the ERL threshold guidelines of 46.7 mg kg^−1^ (US EPA, 2009). However, levels observed in the North zone were considerably lower when comparing pooled site Pb concentrations from previous studies, Ongeri et al. (2009) (62.5 mg kg^−1^), with a fourfold decrease compared to Ochieng et al. (2006) (102.4 mg kg^−1^) and Outa et al. (2020) (100.9 mg kg^−1^). Conversely, Pb concentrations from the South zone were double of those reported by Ongeri et al. (2009) (15.9 mg kg^−1^) and 1.5 times that of Outa et al. (2020) (21.7 mg kg^−1^), but were below the 2006 study by Ochieng et al. (2006) (94.5 mg kg^−1^), with a suggested threefold decrease in this PHE during the last decade and a half. Overall, Pb concentrations from the Lake zone were at the lowest levels of all the zones and for all previous studies. The fall in Pb concentrations could be due to the removal of this metal in Pb-based fuel during the last decade, but persistence may be due to the continued use of Pb-based paints in Kenya, (Kessler, 2014) and more probably a legacy runoff from contaminated soils and open garages (Outa et al., 2020).

In the case of Zn, contributions have shown consistently high levels across the four zones and above the ERL thresholds. Outa et al. (2020) indicated levels of Zn have probably accumulated from increased runoff and wastewater inputs causing sediment enrichment in the Winam Gulf, with this trend continuing and resulting in ERL thresholds being exceeded over the last 15 years, as observed in the present study. These persistently high levels of Zn are a cause of concern and are potentially linked to increased anthropogenic and industrial activities and as a result of inadequate management of waste and sewage disposal facilities around the shores of the Winam Gulf, (Juma et al., 2014). Levels of Cr were found to be four to ten times greater in the North, South and Lake zones since the 2006 study by Ochieng et al. (2006) and remained high compared to Outa et al. (2020) study over a decade later. There has clearly been a sharp increase in concentrations of Cr, with levels now above the TEC thresholds (MacDonald, 2000) for the North, South and Lake zones and above the ERL threshold in the Strait zone during the last 15 years. The continued rise in Cr sediment levels could be attributable to increased alluvial exploration such as mining and sand extraction (Okoth. et al., 2010) and the release of this metal from the surrounding geological bedrock (see URL_3; Watts et al., 2021). This obviously increases the risk of biomagnification and, as a result, the dangers to the fish-eating communities as observed by Velma et al. (2009). Although Cr accumulates less in muscle tissue (the most consumed part of the fish) than in gills and skin (Avenant-Oldewage & Marx, 2000), the risk to fish-consuming communities cannot be underestimated with further research required to understand the risks and to mitigate the effects of this PHE.

Three previous studies (Ongeri et al., 2009; Njiru et al., 2013 and Outa et al., 2020) have suggested that PHEs within surface sediments reflect the limited exchange of metals within Winam Gulf when compared to the main Lake, with increased influxes from industrial (discharges) and anthropogenic (urban) sources being retained in these locations. Indeed, in the results from the present study, the four zones indicate some similarities with concentrations of PHEs in areas where these activities may occur, and are also partially driven by the effects of soil erosion/wash off in the catchment from increased land use/changes from geogenic sources (URL_3; Watts et al., 2021).

#### BAF-BSAF measurements in aquaculture fish

The BAF of observed PHE concentrations in the ratio of fish to water indicated none were observed at bioaccumulation levels in the tissues of the Nile tilapia analysed in the present study (Suppl. Table 4). Two macro-elements Mg and K were observed above the BAF values of > 1000 bioaccumulative and > 5000 extremely bioaccumulative, respectively. Increased levels of K could be attributed to its biological function in the fish rather than as a bioaccumulation effect. Potassium is associated with fish metabolic and physiological processes and maintenance of cellular and intracellular ion volume, nerve and membrane function potentials (Lall & Kaushik, 2021). Levels of Mg (BAF 1141) in the Lake cage fish were slightly above the < 1000 threshold defined as “no probability of bioaccumulation” (Arnot & Gobas, 2006). Magnesium levels may also be attributable to biological and metabolic functions, similar to those defined above for K (Lall & Kaushik, 2021) rather than a probable bioaccumulation effect (BAF > 1000 < 5000). In the remaining zones, (North, South and Strait) levels on both caged and wild fish tissue were below the BAF < 1000 threshold.

The BSAF of observed PHE concentrations in the ratio of fish to sediments Thomann et al. (1995) and Dallinger et al. (1993) were all < 1 with the exception of Hg (BSAF 1.06) in the South zone for wild fish (Suppl. Table 5). The BSAF value was slightly above the deconcentrator threshold (< 1) at 1.06 and defined as a microconcentrator. Additionally, one macro-element (K) for caged fish (Strait and Lake zones) and wild fish (South and Strait zones) and one micro-element (Se) for wild fish (North and South zones) were also identified as micro-concentrators (BSAF > 1—< 2). Values for K for all fish types and all zones were within a 10% range of each other and potentially attributable to natural biological and physiological processes as indicated for BAF rather than a probable BSAF influence. (Lall & Kaushik, 2021; Tacon, 1987). Similarly, Se an essential micronutrient, which when converted from its four inorganic oxidation states to a bioavailable organic seleno-amino acid form, is responsible for numerous and diverse biological functions (Lall & Kaushik, 2021). However, there are implications in its potential to accumulate to levels that are toxic due to a relatively narrow range between toxicity and biological requirements. The effects of excess bioaccumulation of Se in fish tissue could be further compounded by the consumption of these fish and the implications of food security with Se toxicity effects in the consumer (Lemly, 1999).

The contributions of most PHEs calculated using threshold guidelines (WHO, 1996; NAS, 2011; JECFA, 2020, Table 7) were below the PMTI levels in both the caged and wild fish tissue in the four zones. However, two PHEs, Cd and Cr were above the PMTI guidelines, with Pb giving similar results to the maximum allowable intake level of 0.003 mg/kg bw/d for the North, South and Strait zones. Notwithstanding, levels of Cd and Cr were a concern with PMTI values observed above threshold guidelines in all zones and for both caged and wild fish. The levels of Cd were two orders of magnitude lower in the cage fish when compared to their wild counterparts and from the same zones (Table 7). This disparity may be attributable to the differing feeding regimes for both caged and wild fish, with wild fish having a wider feeding niche than their caged counterparts and the bioavailability of this metal. Similarly, Cr levels were variable between cage and wild fish in the four zones with differences ranging between ± 0.2 mg/kg bw/d. Although Cr was observed to be above their PMTI level, the WHO has indicated that our tolerable limit for Cr is quite high (WHO, 1996). This may be in part due to the relative non-toxic nature of Cr when found in food sources, and findings have indicated that additional supplements or Cr on top of the PMTI in certain instances improve hypoglycaemia, impaired glucose tolerance and improve circulating insulin levels and the lipid profile (WHO, 1996). However, changes in the levels of Cr limits are still ongoing and the supplementation of Cr should not exceed the stated guidance level (WHO, 1996). Chromium is particularly toxic to fish in its organic form, which speciates under a range of abiotic conditions within the water environment. The hexavalent (Cr^4+^) is most capable of crossing the biological membranes and thus accumulates easily in tissues of freshwater fishes (Avenant-Oldewage and Marx, 2000) with potential acute and chronic effects. Given that the speciation of Cr in fish tissues is in the order of gills > liver > muscle > skin (Velma et al., 2009), concerns about health risk from the consumption of fish may be lower by the fact that most often, the organs and tissues with higher concentrations of Cr are seldom eaten. However, there is scope for further studies to identify the sources and pathways of Cr, with information from these studies used to put controls in place to mitigate their short- and long-term impacts.

Most if not all the macro-/micronutrient elements for the present study in both the wild and caged Nile tilapia were observed to be below their respected desired RDI concentrations levels, with most falling below 10% of the RDI (NAS, 2011). However, although the dietary consumption of fish does not contribute significantly to macro-/micronutrient supply (see Marriott et al., 2019), one element, Se, differed in that the RDI for all locations and fish type (wild or caged) ranged between 16 and 29% RDI with wild fish being slightly higher in their tissue concentrations of Se than the caged counterparts (Table 6). Similar variations for Se (25–40% RDI) have been observed elsewhere by Marriott et al. (2019) when comparing fish sourced from urban, market and rural fishponds of West Bengal, India. The variability in the Se concentrations for wild tilapia may be a factor of their feeding niche as the wild fish have a more varied diet and a wider feeding area in which to source their prey/food items than their caged counterparts that are fed commercial feeds and are fixed in one location. Additionally, Se (as indicated previously) is a major factor in diverse biological functions, and can be absorbed in small amounts from the aquatic environment via their gills although absorption is primarily via the gastrointestinal tract (Lall & Kaushik, 2021).

There have been numerous studies on bottom sediments over the last three decades highlighting the impact of anthropogenic activities and the discharge of heavy metals including PHEs into Winam Gulf and the wider Lake Victoria (Mwamburi, 2003; Ongeri et al., 2009; Oyoo-Okoth et al., 2010 and references therein; Bakyayita et al., 2019). Areas around the city of Kisumu have seen increased activity and ultimately increases in PHEs as previously described (Ongeri et al., 2009). In that study, metal concentrations indicated a trend of Fe > Zn > Cu > Pb > Cd in all of their sampling locations around Kisumu (including Dunga Beach), with only the River Nyando differing with a reversal of Pb and Cu concentrations (Pb > Cu) in discharge into the surrounding Winam Gulf. The present study indicated a similar trend in that Fe was again the dominant metal with Fe > Zn > Cu > Cr > Ni > Pb. It must be noted that although the North and South zones encompass areas of high anthropogenic activity such as the city of Kisumu and Dunga (North) and high riverine input from the Rivers Nyando and Sondu-Miriu (South), with areas located up these rivers being utilised by local communities, their mean concentrations across these two zones have remained relatively consistent. Aluminium (not reported by Ongeri et al., 2009) was measured at elevated levels within all four zones, with a total combined average of 74,000 mg kg^−1^. Aluminium and other metals identified in the sediments from this study can be indicative of natural (geogenic) sources, such as natural weathering from the surrounding mineralised lithology/geology, or from sorption–desorption processes of these metals onto organic matter and sediment (URL_3; Watts et al., 2021). The high levels of Cu and Pb observed in the North zone could be attributable to increased anthropogenic activities in this locality through uncontrolled industrial waste discharges, such as from Kisumu (Mwamburi, 2003; Ongeri et al., 2009) and through domestic and municipal waste inputs (Ongeri et al., 2009). These instances have been identified as some of the main contributors to the high metal/PHEs into Winam Gulf, exacerbated by the vast river systems that surround the city of Kisumu and wider Winam Gulf (Mwamburi, 2003; Ongeri et al., 2009; Oyoo-Okoth et al., 2010).

Comparing the present water quality zonal data to previous studies in Winam Gulf and Lake Victoria by Ongeri et al. (2009) and Ochieng et al. (2006) and considering the potential impacts on the livelihoods of the local communities utilising Winam Gulf as a natural supply of drinking water and other resources, thresholds levels of some PHE, e.g. Al, could be a concern. Inputs from the rivers surrounding and flowing into the Winam Gulf catchment continue to be a probable source of particulate loading, which subsequently increases their wider impact into the Winam Gulf and main body of the Lake (Awange & Obiero, 2006; Omwoma et al., 2014). Similarly, an increase in urban settlements, combined with industrialisation and agricultural land clearance/use along the shores, could see a general rise in water contamination from PHEs. The impacts of this are seen through increased turbidity and eutrophication effects from erosion in the catchment area (Campbell et al., 2003; Okungu et al., 2005; Okely et al., 2010; Nyamweya et al., 2016; Humphrey et al., 2022). The implications from anthropogenic inputs with regards water quality whether riverine or coastal have the potential to become problematic when considering sustainable aquaculture in the Winam Gulf and for the local communities who manage and rely on these systems for food. However, whilst aquaculture in this region is currently thought to cause no consistent environmental changes and the quality of the fish in this study raise few concerns with regards their quality for consumption, inevitably the discharge of particulates such as uneaten feed, faecal and excretory products (including antibiotics) from aquaculture activity will also need monitoring to assess its long-term effect on the ecosystem of the Lake and ultimately implications for future food security (Aura et al., 2018; Kundu et al., 2017).

## Conclusions and recommendations

The aquaculture industry will undoubtably continue to grow, with cultured fish set to provide a greater proportion of dietary protein in developing countries in the coming decades and acting as a major driver of socio-economic growth under the Sustainable Development Goals (SDGs) (FAO, ). Fish are an excellent source of natural proteins, omega oils and lipids in the diet, and in the present study they are also shown to be a minor source of essential nutrients, particularly Se (URL_3; Watts et al., 2021). Their nutritional quality must also be understood in terms of addressing food sufficiency and micronutrient deficiency termed as “hidden hunger” in that fish could supply up to 10% of the RDI for many essential elements with Se supplying up to 29% of the RDI.

There is evidence of contamination from PHEs across all the four zones surveyed in Winam Gulf and the wider Lake Victoria basin. This is a point of concern, with some persisting or shown to be increasing when compared to the data from studies conducted over the last 15 years. Increases in the population and continued expansion of commercial activities combined with inadequate management of waste disposal facilities have continued the influx of urban/commercial discharge into Winam Gulf and the wider Lake basin. Together with the re-opening in 2020 of the Kisumu Port and railway hub, there is now a concern from increased dredging that could see the possible re-suspension or re-mobilisation of these PHEs into the surrounding water column. If these activities continue, they could lead to secondary contamination, with a potential to bioaccumulate and biomagnify these PHEs into the aquatic food chain. As a result, this has the potential to increase the hazards and the potential human health risk from the consumption of wild and aquaculture fish, especially when considering the levels of Cr, Cd and in some zones Pb observed in this study.

If we are to mitigate against the continued detrimental impacts from increasing anthropogenic activities along the Winam Gulf shores and the wider Lake Victoria basin from these “pollutants”, a strategy of monitoring and management of these toxins and their bioavailability must be put in place. Changes in practice, by introducing policies that can alleviate and encourage better management practices and strengthening of our understanding of these impacts and how to control them are essential in order to protect the sustainability of Winam Gulf and the Lake Victoria basin. Such monitoring should follow the temporal regularity of cultured fish harvesting seasons which will also introduce coincidence of the bi-annual precipitation peak rhythms. Focus should then be made on the critical habitats such as the cage culture dominated areas, river mouths and other known point sources of pollutants, e.g. key fish markets and major landing beaches. Strategies must also include the use of key local resources, including environmental management teams, such as the Lake Victoria Fisheries Organisation, Lake Victoria Basin Commission, the Kenya Marine and Fisheries Research Institute, National Environmental Management Authority and the Kenya Maritime Authority to continue to monitor and make aware the causes of environmental degradation, assist in evaluating their impacts on the Lake and how we address mitigation processes in preventing the loss of this invaluable resource. Such multi-stakeholder, multi-institutional integrated methods offer a coordinated and synergistic approach towards long-term sustainable management. One of the notable cases of best practices is the approach used by the UK Centre for Ecology and Hydrology in management of the Cambrian Lakes and Loch Leven in England and Scotland, respectively (URL_5). Furthermore, for ease of management and control of pollutant streams, there is a need to zone the lake according to sustainability of various uses such as aquaculture as recommended by May et al. (2021). Finally, it is important to create sensitisation channels and drive local and community education on the state of the ecosystem and resource base for future management strategies.

## Supplementary Information

Below is the link to the electronic supplementary material.

Supplementary file1 (DOCX 63 kb)
