# Supplementary material for: Considerations for environmental biogeochemistry and food security for aquaculture around Lake Victoria, Kenya
Source: Environ Geochem Health. 2023 Jun 2;45(8):6137–62. doi: 10.1007/s10653-023-01585-w (PMC10403404; doi:10.1007/s10653-023-01585-w)
Supplement: Supplementary file 1 — Supplementary file1 (DOCX 63 kb) [file 10653_2023_1585_MOESM1_ESM.docx]

Supplementary Table 1. Limits of detection (LOD) are presented for Water (a), Sediment (b) and Fish muscle tissue (c) measurable by ICP-MS. Where indicated * Hg was measured using the DMA-80 analyser.

| **Water (a)** | **Li** | **Be** | **B** | **Na** | **Mg** | **Si** | **Al** | **P** | **S** | **K** |
| --- | --- | --- | --- | --- | --- | --- | --- | --- | --- | --- |
|  | µg L^-1^ | µg L^-1^ | µg L^-1^ | mg Lˉˡ | mg Lˉˡ | mg Lˉˡ | µg L^-1^ | mg Lˉˡ | mg Lˉˡ | mg Lˉˡ |
| **LOD** | 3 | 0.03 | 30 | 0.4 | 0.008 | 12 | 2 | 0.04 | 0.2 | 0.3 |
|  |  |  |  |  |  |  |  |  |  |  |
|  | **Ca** | **Ti** | **V** | **Cr** | **Mn** | **Fe** | **Co** | **Ni** | **Cu** | **Zn** |
|  | mg Lˉˡ | µg L^-1^ | µg L^-1^ | µg l-1 | µg L^-1^ | µg L^-1^ | µg L^-1^ | µg L^-1^ | µg L^-1^ | µg L^-1^ |
| **LOD** | 0.5 | 0.2 | 0.07 | 0.08 | 0.2 | 0.4 | 0.02 | 0.06 | 0.3 | 0.8 |
|  |  |  |  |  |  |  |  |  |  |  |
|  | **Ga** | **As** | **Se** | **Rb** | **Sr** | **Y** | **Zr** | **Nb** | **Mo** | **Ag** |
|  | µg L^-1^ | µg L^-1^ | µg L^-1^ | µg L^-1^ | µg L^-1^ | µg L^-1^ | µg L^-1^ | µg L^-1^ | µg L^-1^ | µg L^-1^ |
| **LOD** | 0.2 | 0.03 | 0.05 | 0.2 | 0.2 | 0.006 | 0.03 | 0.02 | 0.02 | 0.02 |
|  |  |  |  |  |  |  |  |  |  |  |
|  | **Cd** | **Sn** | **Sb** | **Cs** | **Ba** | **La** | **Ce** | **Pr** | **Nd** | **Sm** |
|  | µg L^-1^ | µg L^-1^ | µg L^-1^ | µg L^-1^ | µg L^-1^ | µg L^-1^ | µg L^-1^ | µg L^-1^ | µg L^-1^ | µg L^-1^ |
| **LOD** | 0.009 | 0.02 | 0.007 | 0.04 | 0.2 | 0.006 | 0.003 | 0.003 | 0.006 | 0.005 |
|  |  |  |  |  |  |  |  |  |  |  |
|  |  |  |  |  |  |  |  |  |  |  |
|  | **Ta** | **W** | **Tl** | **Pb** | **Bi** | **Th** | **U** |  |  |  |
|  | µg L^-1^ | µg L^-1^ | µg L^-1^ | µg L^-1^ | µg L^-1^ | µg L^-1^ | µg L^-1^ |  |  |  |
| **LOD** | 0.007 | 0.02 | 0.007 | 0.02 | 0.02 | 0.04 | 0.004 |  |  |  |
|  |  |  |  |  |  |  |  |  |  |  |

| **Sediment (b)** | | **Hg* (DMA)** | | **Li** | | **Be** | | **B** | | **Na** | | **Mg** | | **Si** | | **Al** | | **P** | |
| --- | --- | --- | --- | --- | --- | --- | --- | --- | --- | --- | --- | --- | --- | --- | --- | --- | --- | --- | --- |
|  | | mg kˉˡ | | mg kˉˡ | | mg kˉˡ | | n/a | | mg kˉˡ | | mg kˉˡ | | n/a | | mg kˉˡ | | mg kˉˡ | |
| **LOD** | | 0.002 | | 0.6 | | 0.08 | | n/a | | 45 | | 7 | | n/a | | 9 | | 97 | |
|  | |  | |  | |  | |  | |  | |  | |  | |  | |  | |
|  | | **S** | | **K** | | **Ca** | | **Ti** | | **V** | | **Cr** | | **Mn** | | **Fe** | | **Co** | |
|  | | mg kˉˡ | | mg kˉˡ | | mg kˉˡ | | mg kˉˡ | | mg kˉˡ | | mg kˉˡ | | mg kˉˡ | | mg kˉˡ | | mg kˉˡ | |
| **LOD** | | 1895 | | 25 | | 466 | | 2 | | 0.7 | | 0.7 | | 0.6 | | 15 | | 0.04 | |
|  | |  | |  | |  | |  | |  | |  | |  | |  | |  | |
|  | | **Ni** | | **Cu** | | **Zn** | | **Ga** | | **As** | | **Se** | | **Rb** | | **Sr** | | **Y** | |
|  | | mg kˉˡ | | mg kˉˡ | | mg kˉˡ | | mg kˉˡ | | mg kˉˡ | | mg kˉˡ | | mg kˉˡ | | mg kˉˡ | | mg kˉˡ | |
| **LOD** | | 0.07 | | 0.3 | | 7 | | 0.05 | | 0.09 | | 0.08 | | 0.2 | | 0.1 | | 0.02 | |
|  | |  | |  | |  | |  | |  | |  | |  | |  | |  | |
|  | | **Zr** | | **Nb** | | **Mo** | | **Ag** | | **Cd** | | **Sn** | | **Sb** | | **Cs** | | **Ba** | |
|  | | mg kˉˡ | | mg kˉˡ | | mg kˉˡ | | mg kˉˡ | | mg kˉˡ | | mg kˉˡ | | mg kˉˡ | | mg kˉˡ | | mg kˉˡ | |
| **LOD** | | 0.2 | | 0.2 | | 0.4 | | 0.2 | | 0.03 | | 0.03 | | 0.02 | | 0.009 | | 0.2 | |
|  | |  | |  | |  | |  | |  | |  | |  | |  | |  | |
|  | | **La** | | **Ce** | | **Pr** | | **Nd** | | **Sm** | | **Eu** | | **Gd** | | **Tb** | | **Dy** | |
|  | | mg kˉˡ | | mg kˉˡ | | mg kˉˡ | | mg kˉˡ | | mg kˉˡ | | mg kˉˡ | | mg kˉˡ | | mg kˉˡ | | mg kˉˡ | |
| **LOD** | | 0.03 | | 0.05 | | 0.006 | | 0.05 | | 0.009 | | 0.003 | | 0.006 | | 0.002 | | 0.005 | |
|  | |  | |  | |  | |  | |  | |  | |  | |  | |  | |
|  | | **Ho** | | **Er** | | **Tm** | | **Yb** | | **Lu** | | **Hf** | | **Ta** | | **W** | | **Tl** | |
|  | | mg kˉˡ | | mg kˉˡ | | mg kˉˡ | | mg kˉˡ | | mg kˉˡ | | mg kˉˡ | | mg kˉˡ | | mg kˉˡ | | mg kˉˡ | |
| **LOD** | | 0.002 | | 0.003 | | 0.002 | | 0.007 | | 0.003 | | 0.02 | | 0.01 | | 0.02 | | 0.009 | |
|  | |  | |  | |  | |  | |  | |  | |  | |  | |  | |
|  | | **Pb** | | **Bi** | | **Th** | | **U** | |  | |  | |  | |  | |  | |
|  | | mg kˉˡ | | mg kˉˡ | | mg kˉˡ | | mg kˉˡ | |  | |  | |  | |  | |  | |
| **LOD** | | 0.09 | | 0.2 | | 0.006 | | 0.004 | |  | |  | |  | |  | |  | |
|  | |  | |  | |  | |  | |  | |  | |  | |  | |  | |
| **Fish (c)** | **Hg***  **(DMA)** | | **Li** | | **Be** | | **B** | | **Na** | | **Mg** | | **Si** | | **Al** | | **P** | |  |
|  | mg kˉˡ | | mg kˉˡ | | mg kˉˡ | | mg kˉˡ | | mg kˉˡ | | mg kˉˡ | | mg kˉˡ | | mg kˉˡ | | mg kˉˡ | |  |
| **LOD** | 0.002 | | 0.4 | | 0.02 | | 3 | | 30 | | 0.6 | | 8 | | 0.9 | | 5 | |  |
|  |  | |  | |  | |  | |  | |  | |  | |  | |  | |  |
|  | **S** | | **K** | | **Ca** | | **Ti** | | **V** | | **Cr** | | **Mn** | | **Fe** | | **Co** | |  |
|  | mg kˉˡ | | mg kˉˡ | | mg kˉˡ | | mg kˉˡ | | mg kˉˡ | | mg kˉˡ | | mg kˉˡ | | mg kˉˡ | | mg kˉˡ | |  |
| **LOD** | 7 | | 19 | | 101 | | 0.07 | | 0.09 | | 0.09 | | 0.08 | | 3 | | 0.007 | |  |
|  |  | |  | |  | |  | |  | |  | |  | |  | |  | |  |
|  | **Ni** | | **Cu** | | **Zn** | | **Ga** | | **As** | | **Se** | | **Rb** | | **Sr** | | **Y** | |  |
|  | mg kˉˡ | | mg kˉˡ | | mg kˉˡ | | mg kˉˡ | | mg kˉˡ | | mg kˉˡ | | mg kˉˡ | | mg kˉˡ | | mg kˉˡ | |  |
| **LOD** | 0.02 | | 0.04 | | 0.5 | | 0.008 | | 0.003 | | 0.007 | | 0.05 | | 0.03 | | 0.003 | |  |
|  |  | |  | |  | |  | |  | |  | |  | |  | |  | |  |
|  | **Zr** | | **Nb** | | **Mo** | | **Ag** | | **Cd** | | **Sn** | | **Sb** | | **Cs** | | **Ba** | |  |
|  | mg kˉˡ | | mg kˉˡ | | mg kˉˡ | | mg kˉˡ | | mg kˉˡ | | mg kˉˡ | | mg kˉˡ | | mg kˉˡ | | mg kˉˡ | |  |
| **LOD** | 0.04 | | 0.008 | | 0.007 | | 0.007 | | 0.004 | | 0.008 | | 0.004 | | 0.007 | | 0.04 | |  |
|  |  | |  | |  | |  | |  | |  | |  | |  | |  | |  |
|  | **La** | | **Ce** | | **Pr** | | **Nd** | | **Sm** | | **Eu** | | **Gd** | | **Tb** | | **Dy** | |  |
|  | mg kˉˡ | | mg kˉˡ | | mg kˉˡ | | mg kˉˡ | | mg kˉˡ | | mg kˉˡ | | mg kˉˡ | | mg kˉˡ | | mg kˉˡ | |  |
| **LOD** | 0.002 | | 0.004 | | 0.002 | | 0.004 | | 0.002 | | 0.002 | | 0.002 | | 0.002 | | 0.002 | |  |
|  |  | |  | |  | |  | |  | |  | |  | |  | |  | |  |
|  | **Ho** | | **Er** | | **Tm** | | **Yb** | | **Lu** | | **Hf** | | **Ta** | | **W** | | **Tl** | |  |
|  | mg kˉˡ | | mg kˉˡ | | mg kˉˡ | | mg kˉˡ | | mg kˉˡ | | mg kˉˡ | | mg kˉˡ | | mg kˉˡ | | mg kˉˡ | |  |
| **LOD** | 0.002 | | 0.002 | | 0.002 | | 0.002 | | 0.002 | | 0.005 | | 0.002 | | 0.003 | | 0.003 | |  |
|  |  | |  | |  | |  | |  | |  | |  | |  | |  | |  |
|  | **Pb** | | **Bi** | | **Th** | | **U** | |  | |  | |  | |  | |  | |  |
|  | mg kˉˡ | | mg kˉˡ | | mg kˉˡ | | mg kˉˡ | |  | |  | |  | |  | |  | |  |
| **LOD** | 0.008 | | 0.002 | | 0.002 | | 0.002 | |  | |  | |  | |  | |  | |  |
|  |  | |  | |  | |  | |  | |  | |  | |  | |  | |  |

Supplementary Table 1d. Certified reference material used in the analysis of Hg concentrations in both fish muscle tissue and pond sediment Using DMA-80.

| **CRM** | **Expected**  **mgkg^-1^** | **Type** | **Source** | **n** | **Mean**  **mgkg^-1^** | **% RSD** | **Recovery (%)** |
| --- | --- | --- | --- | --- | --- | --- | --- |
| *Fish Tissue* | |  |  |  |  |  |  |
| ERM – BB442 | 0.601 ±0.082 | Fish muscle | *Pollachius virens* (saithe) | 26 | 0.589 | 14.0 | 98.1 |
| MESS-3 | 0.091 ±0.002 | Marine sediment | Esquimalt Harbour, BC | 14 | 0.091 | 2.7 | 100.5 |
| MESS-4 | 0.080 ±0.004 | Marine sediment | Esquimalt Harbour, BC | 11 | 0.059 | 7.5 | 74.1 |
| TH-2 | 0.620 ±0.037 | Lake sediment | Great Lakes basin, North America | 14 | 0.651 | 5.7 | 105.0 |
| HR-1 | 0.342 ±0.029 | Lake sediment | Great Lakes basin, North America | 12 | 0.301 | 9.7 | 88.1 |
|  |  |  |  |  |  |  |  |
| *Sediment* |  |  |  |  |  |  |  |
| MESS-3 | 0.091 ±0.005 | Marine sediment | Esquimalt Harbour, BC | 8 | 0.093 | 5.8 | 97.9 |
| MESS-4 | 0.080 ±0.069 | Marine sediment | Esquimalt Harbour, BC | 24 | 0.069 | 8.3 | 116.7 |
| TH-2 | 0.620 ±0.057 | Lake sediment | Great Lakes basin, North America | 18 | 0.765 | 7.5 | 80.9 |
| HR-1 | 0.342 ±0.038 | Lake sediment | Great Lakes basin, North America | 25 | 0.362 | 10.6 | 94.5 |
| PACS-2 | 3.040 ±0.159 | Marine sediment | Esquimalt Harbour, BC | 9 | 3.145 | 5.1 | 96.7 |

Supplementary Table 1e. Certified reference material used in the analysis of element concentrations in both fish muscle tissue and pond sediment by ICP-MS.

| **Fish Tissue** |  |  |  |  |  |  |  |  |  | **BCR-627** |  | |  | |
| --- | --- | --- | --- | --- | --- | --- | --- | --- | --- | --- | --- | --- | --- | --- |
| **ERM-BB422** | **Na** | **Mg** | **K** | **Ca** | **Mn** | **Fe** | **Cu** | **Zn** | **As** | **As** | **Se** | | **Cd** | |
|  | mg/kg | mg/kg | mg/kg | mg/kg | mg/kg | mg/kg | mg/kg | mg/kg | mg/kg | mg/kg | mg/kg | | mg/kg | |
| **n=** | 13 | 13 | 13 | 13 | 13 | 13 | 13 | 13 | 13 | 10 | 13 | | 13 | |
| **Certificate value (mg/kg)** | 2800 | 1370 | 21400 | 342 | 0.368 | 9 | 1.670 | 16.0 | 12.7 | 4.80 | 1.330 | | 0.008 | |
| **Average** | 3373 | 1692 | 23956 | 408 | 0.466 | 14 | 1.919 | 19.319 | 13.693 | 5.062 | 1.473 | | 0.009 | |
| **sd** | 333.9 | 142.7 | 2257.8 | 49.2 | 0.062 | 1.75 | 0.232 | 2.78 | 1.58 | 0.52 | 0.201 | | 0.002 | |
| **RSD %** | 9.9 | 8.4 | 9.4 | 12.0 | 13.2 | 12.5 | 12.1 | 14.4 | 11.5 | 10.3 | 13.6 | | 23.0 | |
| **Recovery (%)** | 120 | 123 | 112 | 119 | 127 | 149 | 115 | 121 | 108 | 105 | 111 | | 125 | |
|  |  |  |  |  |  |  |  |  |  |  |  | |  | |
| **Sediment** |  |  |  |  |  |  |  |  |  |  |  | |  | |
| **MESS-4** | **Li** | **Be** | **Na** | **Mg** | **Al** | **P** | **S** | **K** | **Ca** | **Ti** | | **V** | |  |
|  | mg/kg | mg/kg | mg/kg | mg/kg | mg/kg | mg/kg | mg/kg | mg/kg | mg/kg | mg/kg | | mg/kg | |  |
| **n=** | 8 | 8 | 8 | 8 | 8 | 8 | 8 | 8 | 8 | 8 | | 8 | |  |
| **Certificate value (mg/kg)** | 65.3 | 2.09 | 12600 | 15800 | 79100 | 1040 | 1580 | 23800 | 13100 | 3840.000 | | 216.000 | |  |
| **Average** | 65.4 | 2.4 | 13752.7 | 16773.9 | 80164.3 | 1057.3 | 1681.0 | 26178.0 | 14224.1 | 3118.6 | | 223.9 | |  |
| **sd** | 4.5 | 0.4 | 535.0 | 1057.1 | 6826.3 | 53.8 | 566.7 | 1803.2 | 645.2 | 171.7 | | 10.8 | |  |
| **RSD %** | 6.8 | 17.5 | 3.9 | 6.3 | 8.5 | 5.1 | 33.7 | 6.9 | 4.5 | 5.5 | | 4.8 | |  |
| **Recovery (%)** | 100 | 115 | 109 | 106 | 101 | 102 | 106 | 110 | 109 | 81 | | 104 | |  |
|  |  |  |  |  |  |  |  |  |  |  | |  | |  |
|  | **Cr** | **Mn** | **Fe** | **Co** | **Ni** | **Cu** | **Zn** | **As** | **Sr** | **Ag** | | **Cd** | |  |
|  | mg/kg | mg/kg | mg/kg | mg/kg | mg/kg | mg/kg | mg/kg | mg/kg | mg/kg | mg/kg | | mg/kg | |  |
| **n=** | 8 | 8 | 8 | 8 | 8 | 8 | 8 | 8 | 8 | 8 | | 8 | |  |
| **Certificate value (mg/kg)** | 94.3 | 298.0 | 37900 | 13.0 | 42.8 | 32.9 | 147.0 | 21.7 | 132.0 | 0.161 | | 0.280 | |  |
| **Average** | 92.8 | 306.2 | 39926.7 | 13.2 | 45.3 | 32.8 | 152.0 | 21.1 | 130.4 | 0.2 | | 0.3 | |  |
| **sd** | 4.3 | 13.5 | 1629.2 | 0.6 | 2.0 | 1.6 | 8.1 | 0.9 | 6.1 | 0.1 | | 0.0 | |  |
| **RSD %** | 4.6 | 4.4 | 4.1 | 4.8 | 4.4 | 4.8 | 5.3 | 4.4 | 4.7 | 46.0 | | 11.6 | |  |
| **Recovery (%)** | 98 | 103 | 105 | 102 | 106 | 100 | 103 | 97 | 99 | 113 | | 91 | |  |

| **Sediment** |  |  |  |  |  |
| --- | --- | --- | --- | --- | --- |
| **MESS-4** | **Sn** | **Sb** | **Tl** | **Pb** | **U** |
|  | mg/kg | mg/kg | mg/kg | mg/kg | mg/kg |
| **n=** | 8 | 8 | 8 | 8 | 8 |
| **Certificate value (mg/kg)** | 2.35 | 1.07 | 0.85 | 21.5 | 3.4 |
| **Average** | 2.3 | 1.0 | 0.9 | 22.8 | 3.0 |
| **sd** | 0.1 | 0.1 | 0.0 | 0.8 | 0.2 |
| **RSD %** | 6.1 | 8.0 | 2.9 | 3.3 | 5.5 |
| **Recovery (%)** | 99 | 98 | 102 | 106 | 88 |

Suppl. Table 2. Concentrations of selected potentially harmful elements (PHEs) and their descriptive statistics in Winam Gulf and Lake Victoria waters as defined by zone. Water quality guidelines are taken from the US. EPA Human Health Criteria (HHC), National Primary Drinking Water regulations (MCL) and National recommended Aquatic Life Criteria (CCC, CMC) (US EPA, URL_1). All values and guidelines are measured in µg L^-1^. HHC = Human health criteria; MCL = Maximum contamination level; CCC = Criterion continuous concentration; CMC = Criterion maximum concentration. Values above the MCL are highlighted in **bold**.

|  |  | **Ag** | **Al** | **As** | **Cd** | **Cr** | **Cu** | **Ni** | **Pb** | **Zn** |
| --- | --- | --- | --- | --- | --- | --- | --- | --- | --- | --- |
| US. EPA | **HHC** | - | - | 0.018 | - | - | 13000 | 610 | - | - |
|  | **MCL** | - | - | **0.01** | **0.005** | **0.1** | **1.3** | - | **0.015** | 7400 |
| U.S. EPA | **CCC** | - | 87 | 150 | 0.25 | - | 9.0 | 52 | 2.5 | 120 |
|  | **CMC** | 3.2 | 750 | 340 | 2.0 | - | 13 | 470 | 65 | 120 |
|  |  | µg L^-1^ | µg L^-1^ | µg L^-1^ | µg L^-1^ | µg L^-1^ | µg L^-1^ | µg L^-1^ | µg L ^L-1^ | µg L^-1^ |
| **North** | Mean | 0.31 | 851 | **0.42** | **0.01** | **0.52** | **1.4** | 0.64 | **0.36** | 4.0 |
|  | ± 1 SD | 0.56 | 494 | 0.07 | 0.01 | 0.28 | 0.54 | 0.21 | 0.22 | 3.9 |
|  | Max | 2.6 | 3324 | 0.59 | 0.05 | 1.9 | 4.4 | 1.6 | 0.85 | 29 |
|  | Min | 0.02 | 2.0 | 0.18 | 0.01 | 0.08 | 0.30 | 0.26 | 0.02 | 0.80 |
| **South** | Mean | 0.12 | 457 | **0.42** | **0.01** | **0.29** | 1.1 | 0.59 | **0.18** | 2.5 |
|  | ± 1 SD | 0.24 | 382 | 0.28 | <0.001 | 0.19 | 0.46 | 0.42 | 0.09 | 1.5 |
|  | Max | 1.4 | 1820 | 2.7 | 0.02 | 0.90 | 2.9 | 3.6 | 0.35 | 6.5 |
|  | Min | 0.02 | 4.0 | 0.07 | 0.01 | 0.08 | 0.30 | 0.17 | 0.03 | 0.8 |
| **Straits** | Mean | 0.08 | 249 | **0.27** | **0.01** | **0.17** | 0.78 | 0.23 | **0.15** | 4.2 |
|  | ± 1 SD | 0.12 | 238 | 0.04 | 0.01 | 0.15 | 1.3 | 0.15 | 0.38 | 8.5 |
|  | Max | 0.34 | 945 | 0.35 | 0.05 | 0.66 | 9.8 | 0.64 | 2.8 | 57 |
|  | Min | 0.02 | 6.2 | 0.16 | 0.01 | 0.08 | 0.30 | 0.06 | 0.02 | 0.80 |
| **Lake** | Mean | 0.02 | 10 | **0.15** | **0.01** | 0.08 | 0.41 | 0.09 | **0.05** | 4.6 |
|  | ± 1 SD | 0.00 | 12 | 0.05 | <0.001 | <0.001 | 0.45 | 0.14 | 0.08 | 12 |
|  | Max | 0.02 | 46 | 0.23 | 0.02 | 0.08 | 2.4 | 0.71 | 0.34 | 55 |
|  | Min | 0.02 | 2.0 | 0.04 | 0.01 | 0.08 | 0.30 | 0.06 | 0.02 | 0.80 |

Suppl. Table 3. Total mean concentrations of selected elements for macro and micro nutrients and potentially harmful elements (PHEs) observed in the tissues of *O*. *niloticus* for both caged and wild fish collected from four zones within the Winam Gulf (North, South, Strait) and Lake Victoria (Lake). All values are taken from dry tissue weight (mg kg^-1^).

|  | ***North*** | | ***South*** | | ***Strait*** | | ***Lake*** | | |
| --- | --- | --- | --- | --- | --- | --- | --- | --- | --- |
|  | Cage | Wild | Cage | Wild | Cage | Wild | | Cage | Wild |
| n | 23 | 21 | 12 | 13 | 21 | 17 | | 4 | 0 |
| **Macronutrients** |  |  |  |  |  |  | |  |  |
| Na | 2738 ± 458 | 2815 ± 508 | 2462 ± 351 | 2816 ± 393 | 2543 ± 428 | 2343 ± 537 | | 2650 ± 59 | - |
| Mg | 1543 ± 151 | 1533 ± 135 | 1462 ± 107 | 1568 ± 90 | 1518 ± 139 | 1595 ± 112 | | 1534 ± 68 | - |
| K | 21400 ± 1648 | 21574 ± 1698 | 20798 ± 1593 | 21859 ± 1613 | 22379 ± 2261 | 22623 ± 1896 | | 23826 ± 777 | - |
| Ca | 3734 ± 6006 | 1310 ± 2145 | 1159 ± 1080 | 1105 ± 785 | 1978 ± 3204 | 1092 ± 645 | | 1020 ± 466 | - |
| **Micronutrients** |  |  |  |  |  |  | |  |  |
| Cu | 5.3 ± 11 | 2.5 ± 2.1 | 1.7 ± 0.93 | 1.6 ± 0.44 | 3.9 ± 6.1 | 2.0 ± 1.3 | | 3.0 ± 0.19 | - |
| Fe | 21 ± 8.8 | 19 ± 6.7 | 21 ± 5.5 | 20 ± 6.4 | 29 ± 33 | 20 ± 7.8 | | 40 ± 30 | - |
| Mn | 2.4 ± 2.9 | 1.7 ± 2.8 | 1.3 ± 0.34 | 1.8 ± 1.0 | 2.1 ± 1.7 | 1.3 ± 0.31 | | 1.2 ± 0.3 | - |
| Se | 0.45 ± 0.12 | 0.71 ± 0.26 | 0.52 ± 0.05 | 0.79 ± 0.27 | 0.58 ± 0.13 | 0.58 ± 0.19 | | 0.57 ± 0.05 | - |
| Zn | 24 ± 5.3 | 26 ± 5.8 | 26 ± 3.1 | 29 ± 5.6 | 30 ± 5.6 | 25 ± 4.0 | | 31 ± 3.4 | - |
| **Potentially Harmful Elements** |  |  |  |  |  |  | |  |  |
| Ag | 0.004 ± 0.001 | 0.004 ± 0.002 | 0.004 ± 0.001 | 0.01 ± 0.004 | 0.004 ± 0.001 | 0.004 ± 0.001 | | 0.004 ± 0.001 | - |
| Al | 3.9 ± 1.7 | 6.0 ± 5.0 | 6.0 ± 3.1 | 5.8 ± 4.2 | 9.3 ± 25 | 5.0 ± 2.9 | | 2.9 ± 0.6 | - |
| As | 0.07 ± 0.08 | 0.05 ± 0.03 | 0.05 ± 0.01 | 0.46 ± 1.43 | 0.03 ± 0.01 | 0.06 ± 0.04 | | 0.13 ± 0.08 | - |
| Cd | 0.002 ± 0.001 | 0.002 ± 0.001 | 0.002 ± 0.001 | 0.002 ± 0.001 | 0.002 ± 0.001 | 0.002 ± 0.001 | | 0.002 ± 0.001 | - |
| Cr | 0.36 ± 0.48 | 0.56 ± 0.88 | 0.55 ± 0.86 | 0.24 ± 0.18 | 0.48 ± 0.73 | 0.23 ± 0.29 | | 0.05 ± 0.001 | - |
| Hg | 0.02 ± 0.01 | 0.03 ± 0.02 | 0.02 ± 0.02 | 0.03 ± 0.01 | 0.04 ± 0.02 | 0.02 ± 0.01 | | 0.01 ± 0.001 | - |
| Ni | 0.19 ± 0.22 | 0.27 ± 0.41 | 0.26 ± 0.39 | 0.19 ± 0.10 | 0.24 ± 0.34 | 0.15 ± 0.20 | | 0.07 ± 0.03 | - |
| Sn | 0.47 ± 1.1 | 0.12 ± 0.20 | 0.05 ± 0.08 | 0.03 ± 0.04 | 0.20 ± 0.42 | 0.07 ± 0.10 | | 0.12 ± 0.07 | - |
| Pb | 0.03 ± 0.05 | 0.02 ± 0.02 | 0.02 ± 0.01 | 0.03 ± 0.03 | 0.02 ± 0.02 | 0.02 ± 0.01 ± | | 0.01 ± 0.01 | - |

**NB**. No wild fish were captured or collected from sites in the Lake Zone.

Suppl. Table 4. The bioaccumulation factors calculated using concentrations in the fish tissue for both wild and caged reared Nile tilapia (*O*. *niloticus*) and the elemental concentrations in water from the four zones in the Winam Gulf and Lake Victoria.

**BAF = Cn _Fish_ / Cn _Water_**

|  |  | **Potentially Harmful Elements** | | | | | | | | | **Macro / Micro Elements** | | | | | |
| --- | --- | --- | --- | --- | --- | --- | --- | --- | --- | --- | --- | --- | --- | --- | --- | --- |
| **Fish Type** | **Zone** | **As** | **Cr** | **Cd** | **Cu** | **Mn** | **Ni** | **Pb** | **Sn** | **Tl** | **Ca** | **Fe** | **K** | **Mg** | **Se** | **Zn** |
| Cage | North | 0.174 | 0.886 | 0.183 | 4.36 | 0.147 | 0.330 | 0.108 | 9.71 | 2.21 | 455 | 0.033 | 3910 | 550 | 7.80 | 8.10 |
| Cage | South | 0.106 | 1.59 | 0.219 | 1.43 | 0.053 | 0.469 | 0.066 | 1.67 | 1.95 | 155 | 0.041 | 5248 | 580 | 9.23 | 9.85 |
| Cage | Strait | 0.105 | 3.22 | 0.179 | 4.45 | 0.587 | 1.06 | 0.134 | 7.74 | 4.47 | 314 | 0.138 | 6225 | 625 | 11.6 | 8.41 |
| Cage | Lake | 1.41 | 0.563 | 0.222 | 10.1 | 0.373 | 1.12 | 0.515 | 5.98 | 1.29 | 320 | 4.60 | 13113 | 1141 | 11.5 | 14.3 |
| Wild | North | 0.129 | 1.37 | 0.201 | 2.02 | 0.102 | 0.474 | 0.092 | 2.50 | 3.59 | 160 | 0.030 | 3942 | 546 | 12.4 | 8.49 |
| Wild | South | 1.06 | 0.700 | 0.240 | 1.28 | 0.074 | 0.335 | 0.110 | 1.03 | 3.58 | 148 | 0.039 | 5516 | 623 | 14.1 | 10.8 |
| Wild | Strait | 0.202 | 1.56 | 0.179 | 2.28 | 0.363 | 0.667 | 0.101 | 2.83 | 3.11 | 174 | 0.097 | 6293 | 656 | 11.7 | 7.17 |

Suppl. Table 5. The bioaccumulation factors calculated using concentrations in the fish tissue for both wild and caged reared Nile tilapia (*O*. *niloticus*) and the elemental concentrations in sediment from the four zones in the Winam Gulf and Lake Victoria.

**BASF = Cn _Fish_ / Cn _Sediments_**

|  |  | **Potentially Harmful Elements** | | | | | | | | | | **Macro / Micro Elements** | | | | | |
| --- | --- | --- | --- | --- | --- | --- | --- | --- | --- | --- | --- | --- | --- | --- | --- | --- | --- |
| **Fish Type** | **Zone** | **As** | **Cr** | **Cd** | **Cu** | **Mn** | **Ni** | **Pb** | **Hg** | **Sn** | **Tl** | **Ca** | **Fe** | **K** | **Mg** | **Se** | **Zn** |
| Cage | North | 0.028 | 0.006 | 0.009 | 0.154 | 0.002 | 0.006 | 0.001 | 0.488 | 0.135 | 0.026 | 0.272 | 0.000 | 0.971 | 0.226 | 0.762 | 0.186 |
| Cage | South | 0.008 | 0.008 | 0.007 | 0.038 | 0.001 | 0.006 | 0.001 | 0.599 | 0.010 | 0.031 | 0.068 | 0.000 | 0.982 | 0.227 | 0.839 | 0.172 |
| Cage | Strait | 0.009 | 0.009 | 0.008 | 0.057 | 0.001 | 0.007 | 0.001 | 0.809 | 0.053 | 0.087 | 0.088 | 0.000 | 1.27 | 0.131 | 0.833 | 0.174 |
| Cage | Lake | 0.055 | 0.000 | 0.010 | 0.041 | 0.001 | 0.002 | 0.001 | 0.114 | 0.038 | 0.024 | 0.026 | 0.000 | 1.51 | 0.085 | 0.751 | 0.217 |
| Wild | North | 0.021 | 0.009 | 0.010 | 0.071 | 0.002 | 0.009 | 0.001 | 0.814 | 0.035 | 0.042 | 0.095 | 0.000 | 0.979 | 0.224 | 1.21 | 0.195 |
| Wild | South | 0.077 | 0.004 | 0.008 | 0.035 | 0.001 | 0.005 | 0.001 | 1.06 | 0.006 | 0.057 | 0.065 | 0.000 | 1.03 | 0.244 | 1.28 | 0.189 |
| Wild | Strait | 0.018 | 0.004 | 0.008 | 0.029 | 0.001 | 0.005 | 0.001 | 0.423 | 0.019 | 0.060 | 0.049 | 0.000 | 1.29 | 0.138 | 0.837 | 0.149 |
